# Pharmacological use of a novel scaffold, anomeric *N*,*N-*diarylamino tetrahydropyran: molecular similarity search, chemocentric target profiling, and experimental evidence

**DOI:** 10.1038/s41598-017-12082-3

**Published:** 2017-10-02

**Authors:** Arramshetti Venkanna, Oh Wook Kwon, Sualiha Afzal, Cheongyun Jang, Kyo Hee Cho, Dharmendra K. Yadav, Kang Kim, Hyeung-geun Park, Kwang-Hoon Chun, Sun Yeou Kim, Mi-hyun Kim

**Affiliations:** 1 Gachon Institute of Pharmaceutical Science & Department of Pharmacy, College of Pharmacy, Gachon University, 191 Hambakmoeiro, Yeonsu-gu, Incheon, Republic of Korea; 2 Natural F&P Corp. 152 Saemal-ro, Songpa-gu, Seoul, Korea; 3 Research Institute of Pharmaceutical Science and College of Pharmacy, Seoul National University, Seoul, Republic of Korea

## Abstract

Rational drug design against a determined target (disease, pathway, or protein) is the main strategy in drug discovery. However, regardless of the main strategy, chemists really wonder how to maximize the utility of their new compounds by drug repositioning them as clinical drug candidates in drug discovery. In this study, we started our drug discovery “from curiosity in the chemical structure of a drug scaffold itself” rather than “for a specific target”. As a new drug scaffold, anomeric diarylamino cyclic aminal scaffold 1, was designed by combining two known drug scaffolds (diphenylamine and the most popular cyclic ether, tetrahydropyran/tetrahydrofuran) and synthesized through conventional Brønsted acid catalysis and metal-free α-C(sp^3^)–H functionalized oxidative cyclization. To identify the utility of the new scaffold 1, it was investigated through 2D and 3D similarity screening and chemocentric target prediction. The predicted proteins were investigated by an experimental assay. The scaffold 1 was reported to have an antineuroinflammatory agent to reduce NO production, and compound 10 concentration-dependently regulated the expression level of IL-6, PGE-2, TNF-α, ER-β, VDR, CTSD, and iNOS, thus exhibiting neuroprotective activity.

## Introduction

In drug discovery, one of the important roles of a synthetic chemist is the rational design of novel drug scaffolds with a high selectivity and promising activity, and another important role is to synthesize them. Although drug discovery starts with a target disease (phenotype-based drug discovery) or a target molecule (target-based drug discovery), chemists are interested in the possible uses of their synthesized molecules. Since the 21^st^ century, synthetic organic chemists or medicinal chemists have strongly studied useful approaches for which compounds should be synthesized and how to synthesize them^1–4^. Using these approaches, although the therapeutic potential of privileged scaffolds has been well investigated, it is still rare to select unprecedented scaffolds (with structural novelty) as a starting point of *in silico* target fishing due to insufficient clues or evidence on plausible targets^5–7^. Therefore, currently, it is neither easy nor efficient for chemists to start drug discovery “from curiosity in chemical structure”. Despite the inefficiency, drug discovery driven by the novelty of a drug scaffold can compensate the general approach driven by a specific target to broaden the drug space of artificial drugs^8^.

**Figure 1:**
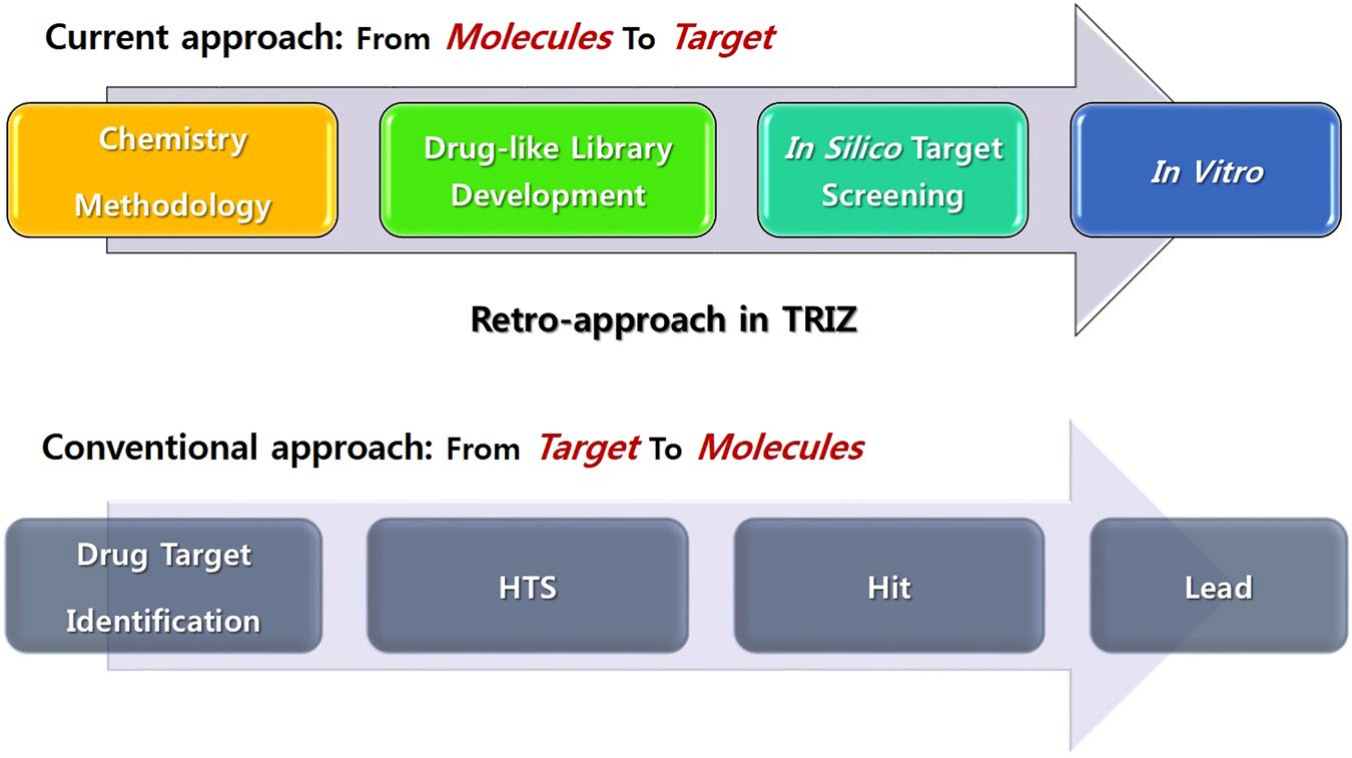
Description on the concept of Chemistry-oriented synthesis (CHOS) through the comparison with conventional target-based drug discovery.

Moreover, it is not recommended to synthesize the unprecedented scaffold without the data on the application of scaffold. But if an organic chemist expands an efficient reaction method for synthesizing an unprecedented scaffold, he is subjected to doubts and questionnaire such like ‘why his products are important and meaningful’, ‘how much different their methodology is with previous studies of other’, and so on. Synthetic chemist tend to judge an unique chemistry rather than unique product for more valuable results. Therefore, the direct translated research between a reaction methodology of unprecedented scaffolds and drug discovery is required to increase the chemical space coverage of artificial drugs. For the same reason, we devised the strategy ‘CHOS (Chemistry oriented synthesis)’ which could possess biological usage from synthetic methodology (Fig. 1) and it is different with current existing strategies: DOS (Diversity oriented synthesis)^1^, BIOS (Biology oriented synthesis)^2^, FOS (Function oriented synthesis)^3^ and TOS (Target oriented synthesis).

**Figure 2:**
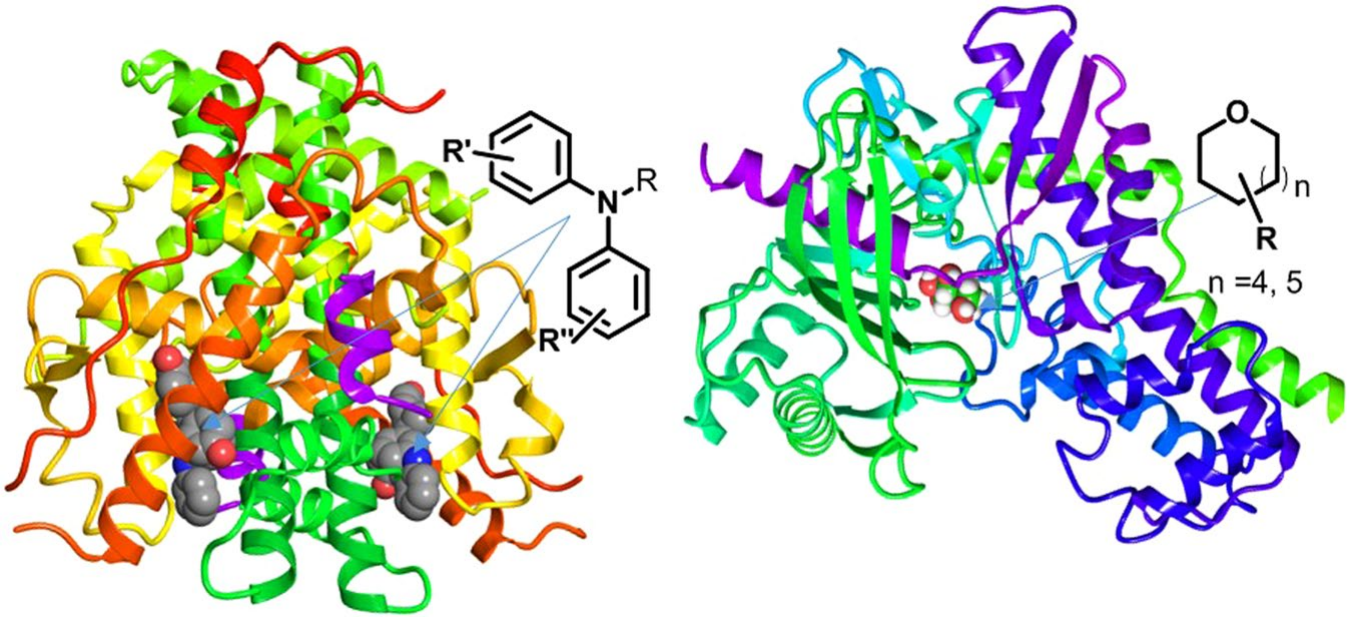
Binding complex of drug scaffolds with their biological targets (left: oestrogen receptor and *N*,*N-*diarylamino, right: selectin and tetrahydropyran).

Diphenylamine is a reported drug scaffold, the skeleton is a suitable core structure for ER ligands, as well as for RAR, RXR, and AR ligands (Fig. 2; left)^9,10^. Tetrahydropyran is also a well-known drug scaffold as well as a substructure of nutrients such as glucose or galactose. In particular, tetrahydropyran is a classical substructure for glycomimetics for the inhibition of proteins (ex. selectins) binding to sugar moieties (Fig. 2; right)^11^. A combined structure of diphenylamine with tetrahydropyran can have any biological utility. However, anomeric diarylamino cyclic aminals such as *N*,*N-*diarylamino tetrahydropyran have not been considered in drug discovery as well as sugar mimetics. Recently, as a part of the CHOS project, we have studied the trilogy on the unprecedented scaffold **1** (synthetic novelty, mechanism, and biological utility) and reported the synthetic methodology of the unprecedented scaffold **1** ^12^ and mechanistic pathway of the metal-free α-C(sp^3^)–H functionalized oxidative cyclization^13^. In this study, we try to report the third part of the trilogy describing the unprecedented scaffold **1**.

In this study, our starting point is the selected unprecedented scaffold **1** without any known target (target molecule, target disease, target pathway, etc.), we couldn’t use structure-based approaches (generally, ‘after choosing molecular target, getting PDB’)^14,15^. In addition, we also couldn’t use general ligand-based approaches (generally, ‘after molecular target, getting active molecules’), which tend to modify structure of active molecules (a template) to get a new drug or to build in silico predictive model for the hit/lead screening^16,17^. Instead of two approaches, we intend to investigate the biological utility of the new scaffold **1** through molecular similarity calculation and chemo-centric target prediction.

## Chemocentric Target Profiling

The chemocentric approach assumes that two similar molecules probably have similar properties; thus, they share the same biological targets or show a similar pharmacological profile^18^. Although a subtle change of one substituent in a drug structure can exceptionally and dramatically change the drug profile (showing an activity cliff), we heuristically know that the drugs in the same drug class (sharing four depths in the five-level classification of ATC code) have structural similarity and the same drug effects^19^. Therefore, the chemocentric approach utilizes the information about compounds (grouped by molecular target; in other words, according to the ligand of protein) instead of the structure of biological targets. This approach has been used for drug repositioning or omics data analysis in the field of polypharmacology and systems biology^20,21^. Chemicals with known activity profile as well as structure have been used to propose new relationships between specific nodes (e.g., the relationship between a disease and specific protein/gene/drug or between a protein and specific protein/drug/metabolite) in this approach. Theoretically, the approach for known drugs can be extended to target prediction for new molecules^22^. However, if structure of a new molecule is very novel, similar compounds are rare, and structural similarity with the new molecule is lower than known drugs; therefore, inferring the biological target of the molecule is more difficult because it is far from the chemocentric assumption. In this study, ‘chemocentric target profiling’, the terminology means the linking of query drug with target profiles of known similar drugs.

## Results

### Chemistry of scaffold 1

**Figure 3:**
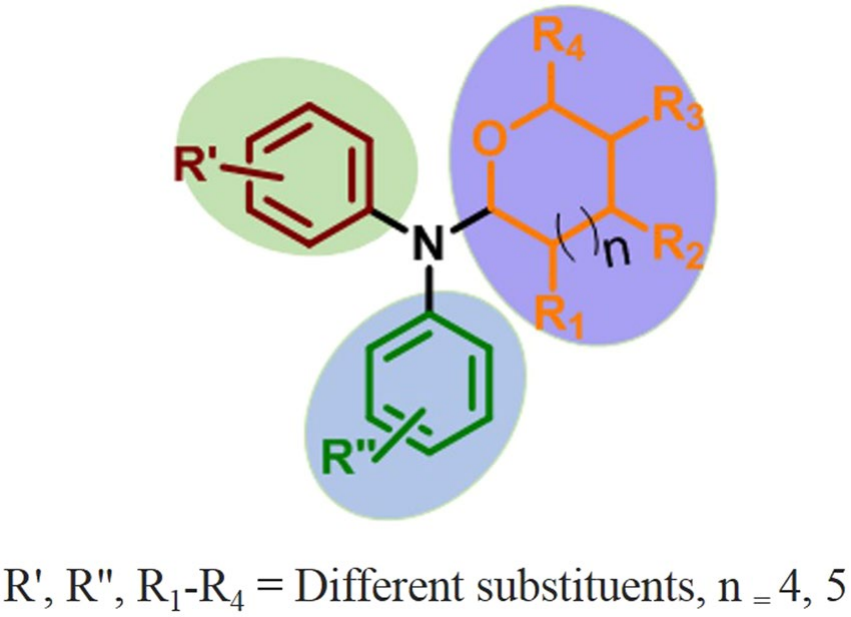
Basic structure of *N*,*N-*diarylamino tetrahydropyran/furan scaffold **1**.

According to our previous study, starting from α-C(sp^3^)–H functionalized amino alcohols, an *N*,*N*-diarylamino tetrahydropyran/tetrahydrofuran scaffold (scaffold **1**) was constructed through α-C(sp^3^)−O bond formation with the loss of only two “H” atoms^12,13^. The structural diversity of the anomeric scaffold **1** has been investigated in the previous study (Fig. 3).

**Figure 4:**
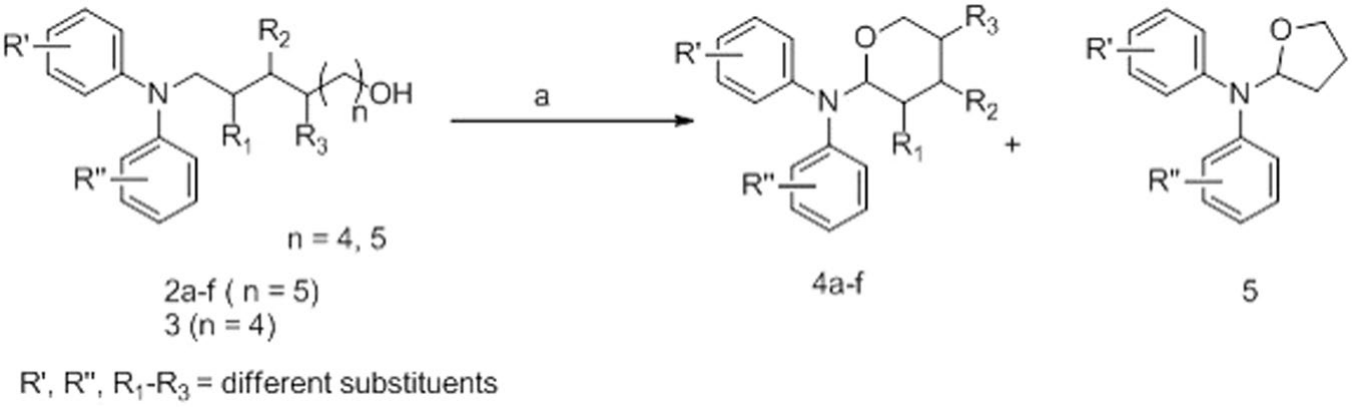
General method for synthesis of scaffold **1**. Reagents and conditions: (a) TEMPO, I_2_, sat. NaHCO_3_/CHCl_3_ (1:3) dark, 0 °C to rt, 1 h.

As shown in Fig. 4, the synthesis was started using tertiary amino alcohols (**2a–f and 3**) prepared by two sequential reactions, amide formation followed by LAH reduction^23,24^ or reductive amination of D-arabinose with bis(4-methoxyphenyl)amine (**2d**)^25^. Then, the tertiary amino alcohols were subjected to free radical TEMPO (2,2,6,6-tetramethyl-1-piperidinyloxy)/iodine-mediated oxidative cyclization reaction^12^, affording the corresponding cyclized compounds (**4a–f** and **5**).

**Figure 5:**
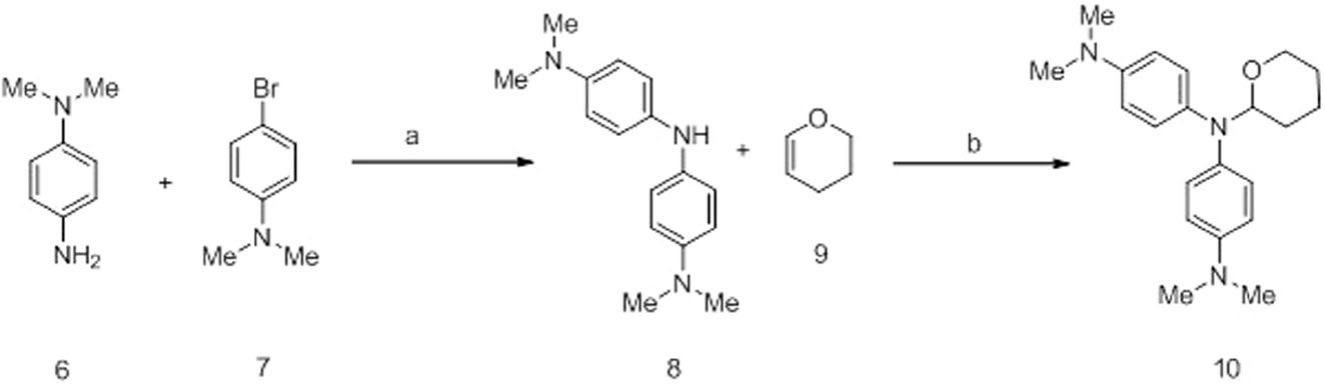
Alternative synthetic method for synthesis of compound **10**. Reagents and conditions: (a) Pd_2_(dba)_3_, XPhos, NaO^t^Bu, anhydrous toluene, rt to 100 °C, 92%; (b) TFA, Et_2_O, 0 °C to rt, 8 h, 65%.

Compound **10** was synthesized via Pd-mediated Buchwald–Hartwig reaction^26^ using readily available starting materials *N* ^1^,*N* ^1^-dimethylbenzene-1,4-diamine (**6**) and 4-bromo-*N*,*N*-dimethylaniline (**7**) followed by Brønsted acid mediated cyclization^27^ with 3,4-dihydro-2*H*-pyran (**9**) (Fig. 5).

### 2D-Fingerprint-based similarity search of scaffold 1

**Figure 6:**
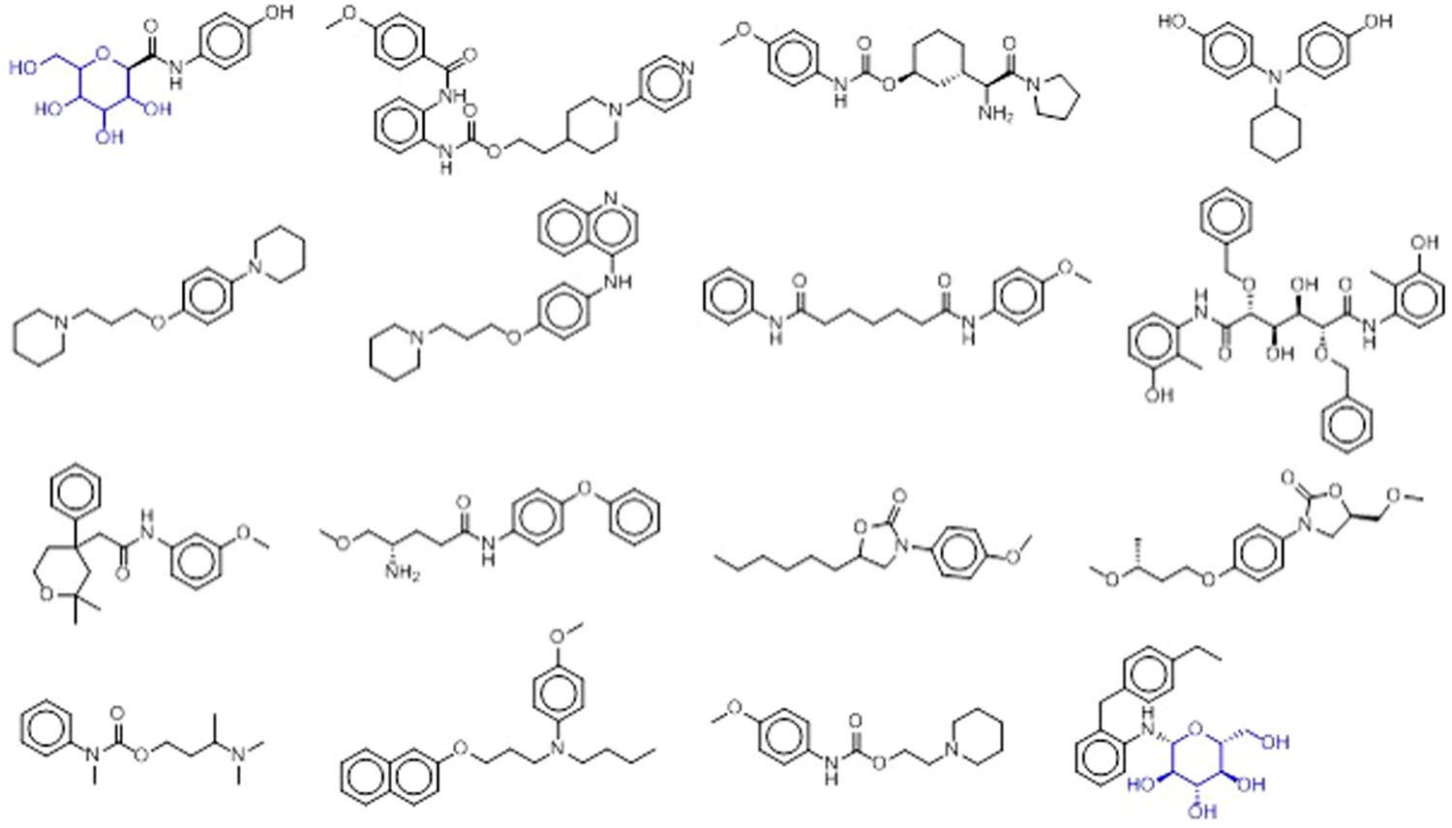
Collection of biologically active compounds similar to scaffold **1** resulting from the 2D fingerprint-based similarity search.

To investigate the target profiling of drug scaffold **1**, first a 2D-fingerprint based similarity search was performed using CSNAP (Chemical Similarity Network Analysis Pulldown)^20^, SIMCOMP (SIMilar COMPound)^28^, and SEA (Similarity ensemble approach)^18^. The CSNAP search showed a total of 109 similar compounds with more than 16 plausible targets (isoprenylcysteine carboxyl methyltransferase, brain glycogen phosphorylase, histone deacetylase, metabotropic glutamate receptor 2, histamine receptor, and sodium/glucose cotransporter 2) as shown in Table 1. In the view of medicinal chemistry, most of the structures of similar ligands in the target list are very different from the structure of scaffold **1** (Fig. 6). Only entries 1, 5, and 7 showed the presence of a tetrahydropyran moiety with a substituent in the 2 position. Entry 14 showed a high topological similarity with scaffold **1**.

**Table 1:** Plausible target profiling of drug scaffold 1 resulting from 2D-similarity based chemocentric network, CSNAP.

No.	Representative Structures	ChEMBL_ID	Target Protein	CTD Gene Symbol	Max T	Frequency
1		CHEMBL105049	Brain glycogen phosphorylase	PYGB	0.635	5
2		CHEMBL1095750	Metabotropic glutamate receptor 2	GRM2	0.618	2
3		CHEMBL1221817	Histone deacetylase	HDA	0.617	2
4		CHEMBL15153	Histamine H1/H2/H3 receptor, Histamine *N*-methyltransferase	HRH, HNMT	0.680	4
5		CHEMBL1800404	Isoprenylcysteine carboxyl methyltransferase	ICMT	0.692	16
6		CHEMBL1822855	HERG, Histamine H3 receptor	KCNH, HRH3	0.667	4
7		CHEMBL2430315	Sodium/glucose cotransporter 2	SLC5A2	0.623	1
8		CHEMBL262464	Human immunodeficiency virus type 1 protease	POL	0.623	4
9		CHEMBL271392	Peroxisome proliferator-activated receptor α, δ, γ	PPAR	0.646	5
10		CHEMBL33295	Monoamine oxidase A	MAOA	0.623	2
11		CHEMBL352737	Dipeptidyl peptidase II, IV, Prolyl endopeptidase	DPF, PREP	0.603	2
12		CHEMBL477276	Leukotriene A4 hydrolase	LTA4H	0.600	2
13		CHEMBL479623	Neuronal acetylcholine receptor protein alpha-4/7/3 subunit	CHRNA	0.609	1
14		CHEMBL495707	Oestrogen receptors α and β	ESR	0.600	1
15		CHEMBL52167	Serotonin 4 (5-HT4) receptor	HTR4	0.660	9
16		CHEMBL66093	Coagulation factor X	F10	0.645	2

In the similarity search using SIMCOMP, the result showed less similarity than that of CSNAP search because only one molecule was allowed in the query. In the similarity search using SEA, the maximum similarity between the ligands of each target protein and our query was <0.4 as shown in Table 2.

**Table 2:** Plausible target profiling of drug scaffold 1 resulting from 2D-similarity search, SEA.

No.	#Lig	Reference Name	E-value	MaxT	CTD Gene Symbol	Diseases	Molecules
1	18	Quinone reductase 1 (human)	0.232	0.30	NQO1	Cytoprotection; cancer; Alzheimer’s disease; tardive dyskinesia	
2	139	Epoxide hydratase (mouse)	0.939	0.35	EPHX	Acute inflammation; MI(Cardiac); Cancer; depression	
3	24	Calpain 2 (pig)	1.25	0.31	CAPN2	Alzheimer’s disease	
4	30	Calpain 2	3.98	0.31	CAPN2	Alzheimer’s disease	
5	30	Quinone reductase 1	4.18	0.30	NQO1	Cytoprotection; cancer; Alzheimer’s disease; tardive dyskinesia	
6	118	Epoxide hydratase	8.31	0.35	EPHX	Acute inflammation; MI(Cardiac); Cancer; depression	

In addition, when the targets generated from the 2D-similarity search were considered, each target could be null hypotheses (H0: There is not the more plausible target molecule of scaffold **1** than the target) to be supported or rejected. E-value represents the possibility to find another target with a higher confidence, and it is difficult to use an E-value of >0.00001 to determine a plausible target. Each E-value of scaffold **1** was >0.001, therefore, it was impossible to achieve a reasonable target profiling. In addition, the three predictions did not provide any consensus result. In sequence, CTD (comparative toxicogenomics database) gene symbols of targets were connected with related diseases, KEGG/Reactome pathways, or target classes (Tables 1 and 2). In the view of potential therapeutic application and mode of action, ‘inflammation’ presented the highest frequency. ‘Cancer’ and ‘neuronal disease’ showed high frequency (Supplementary Table 1).

### Inhibitory effect on lipopolysaccharide (LPS)-induced nitric oxide (NO) production and cell viability assay of scaffold 1

The effects of compounds (**4a–f**, **5**, and **10**) on LPS-induced NO production were investigated in BV-2 microglial cells; 6-Shogaol was used as a known positive control^29^. The observed IC_50_ values are summarized in Table 3

**Table 3:** Inhibitory effects of compounds (**4a–f**, **5**, and **10**) on LPS-induced NO production in BV-2 cells.

Entry	Compound	Structure	Yield (%)	IC_50_(µg/mL)^a^		Entry	Compound	Structure	Yield (%)	IC_50_(µg/mL)^a^
**1**	4a		70	75.66		6	4f		78	20.21
**2**	4b		89	>500		7	5		31	>500
**3**	4c		45	162.49		8	10		65	12.57
**4**	4d		80	>500		9	6-Shogaol^b^		-	5.59
**5**	4e		99	>500						

^a^Data are presented as means ± of three independent experiments. ^b^Positive control.

.

**Figure 7:**
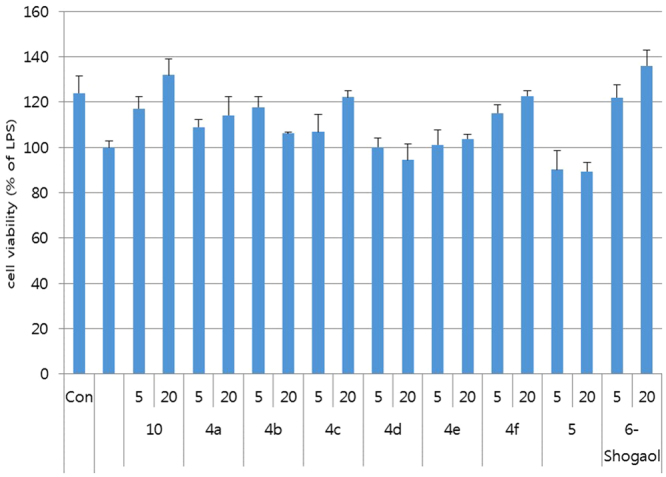
Cytoprotective effect of compounds (**5**, **4a–f**, and **10**) on BV-2 cell lines. The BV2 cells were treated with 100 ng/mL of LPS 30 min after the compounds were treated for 24 h. Cell viability was expressed as a percentage of LPS-treated cells (set as 100%). All the data are presented as the mean ± SEM of three independent experiments. *p < 0.05, **p < 0.01, ***p < 0.001 vs. LPS-treated cells.

The results show that compound **10** exhibited a significant inhibitory activity on NO production with an IC_50_ of 12.57 µM, and compound **4f** exhibited a moderate inhibition of NO production with an IC_50_ of 20.21 µM. Other compounds (**4a–e** and **5**) did not show an effective inhibitory activity to suppress NO production. Along with the NO assay, the cell viability of compounds was measured in LPS-treated microglial BV2 cells to evaluate cytotoxicity (Fig. 7).

After the treatments at two concentrations (5 and 20 μM) of compounds (**4a–f**, **5**, and **10**) on LPS-induced BV2 cells, the tested compounds except for compounds **4d**, **4e**, and **5** at least overcame the LPS-induced cell toxicity in one concentration. In particular, compounds **4a**, **4c**, **4 f**, and **10** concentration-dependently increased the cell viability as a protective agent, consistent with the inhibitory activity of NO production. Among them, compound **10** exhibited the most outstanding antineuroinflammation activity in LPS-induced BV2 cells.

### Effect of compound 10 on LPS-induced TNF-α, IL-6, and PGE2 production in BV-2 cells

**Figure 8:**
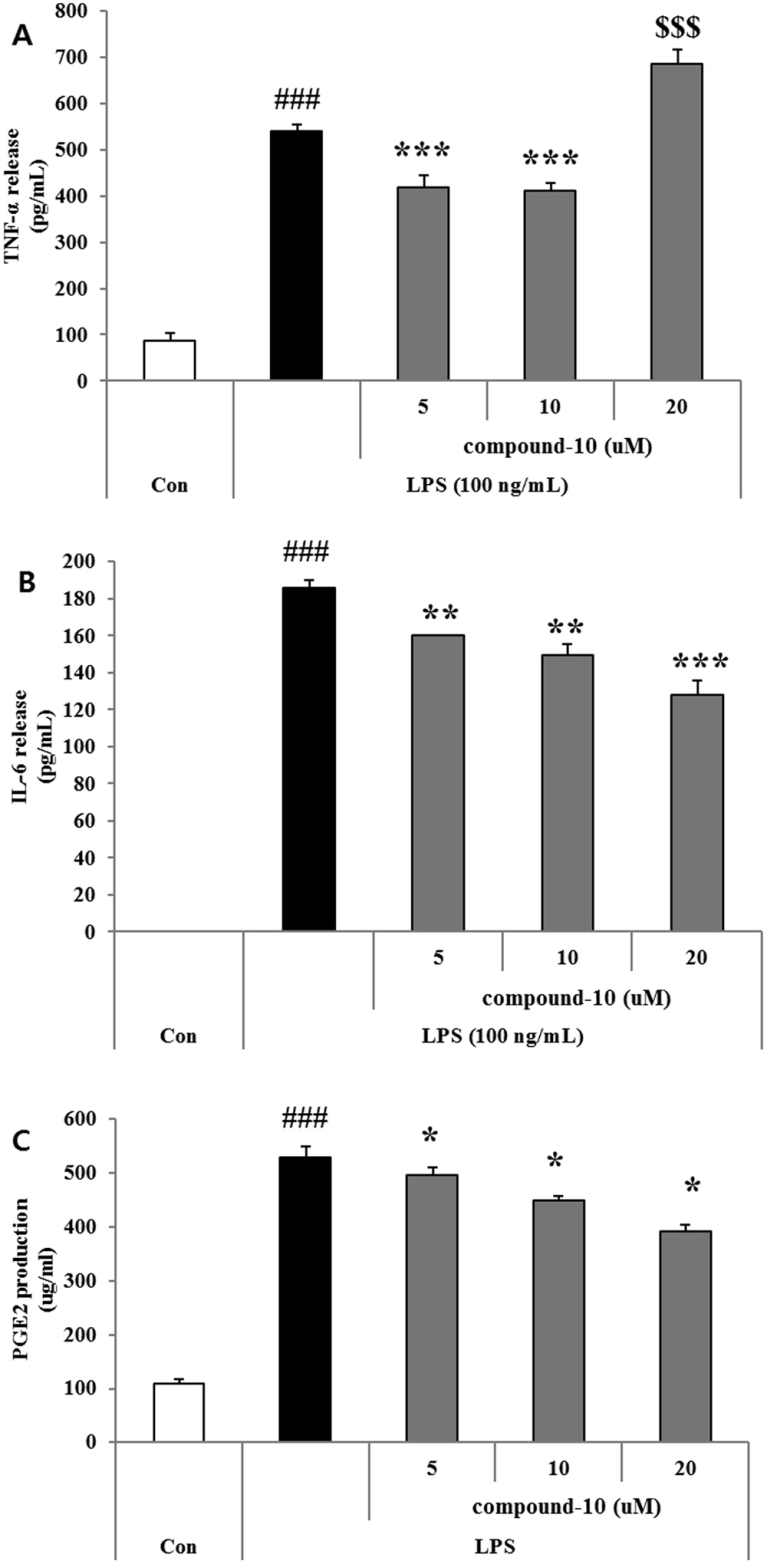
Inhibitory effect of compound 10 on the production of inflammatory mediators in LPS-stimulated BV-2 cells. BV-2 cells were treated with 100 ng/mL of LPS for 30 min and then treated with compound 5 for 24 h. (**A**–**C**) Effect of compound **10** on proinflammatory cytokines, TNF-α, IL-6, and PGE2 in LPS-stimulated BV-2 microglia cells. PGE2 was measured using a competitive immunoassay kit (Cayman Chemical, Ann Arbor, MI, U.S.A). TNF-α and IL-6 were measured using an ELISA development kit. All the data are presented as the mean ± SEM of three independent experiments. *p < 0.01, ***p < 0.001 vs. LPS-treated cells.

LPS induces inflammation via the up-regulation of proinflammatory mediators such as NO, PGE2, and COX2, and proinflammatory cytokines including IL-6 and TNF-α^30^. Thus, the anti-inflammatory effect of compound **10** on the expression level of proinflammatory mediators and cytokines was further investigated. At 10 μM, compound **10** reduced TNF-α by 30%, IL-6 by 22%, and PEG2 by 10% compared to the negative control treated with LPS (Fig. 8). Although the production of IL-6 and PEG2 was concentration-dependently regulated by compound **10**, the TNF-α levels decreased and increased in the pre-to-post comparison of 10 μM.

### Shape-based 3D-similarity search and chemocentric target profiling

**Figure 9:**
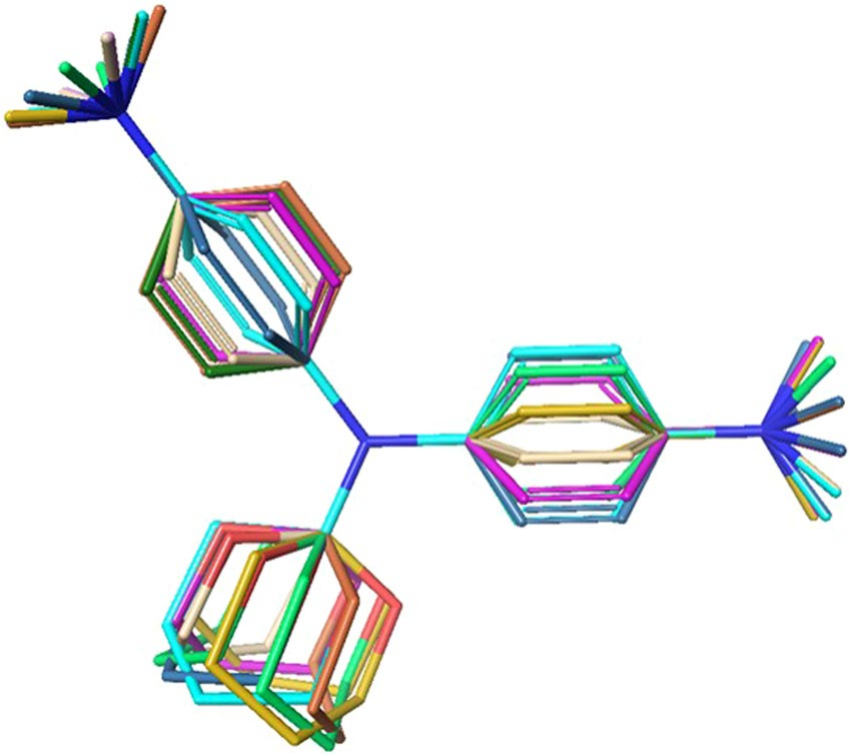
Dynamic tree cut algorithm conformer ensemble of compound **10** for 3D shape-based similarity search.

3D-similarity screening between scaffold **1** (query) and ChEMBL (database) was calculated through the overlap of atomic Gaussians function using ROCS^31–33^. Among the tested compounds, the most efficient compound **10** in suppressing neuroinflammation was selected as the representative of scaffold **1**; therefore, the representative compound was considered as the best query in the 3D search. The conformations of both the query and database should be considered before the 3D-similarity search. To perform a reasonable sampling of conformers in the query and to ensemble conformers with the optimal number of conformers, our recent resampling method of representative conformer ensemble using shape-based alignment and dynamic tree cut algorithm was used^34^. After the resampling, multi-conformers of compound **10** were used as the query (Fig. 9).

**Figure 10:**
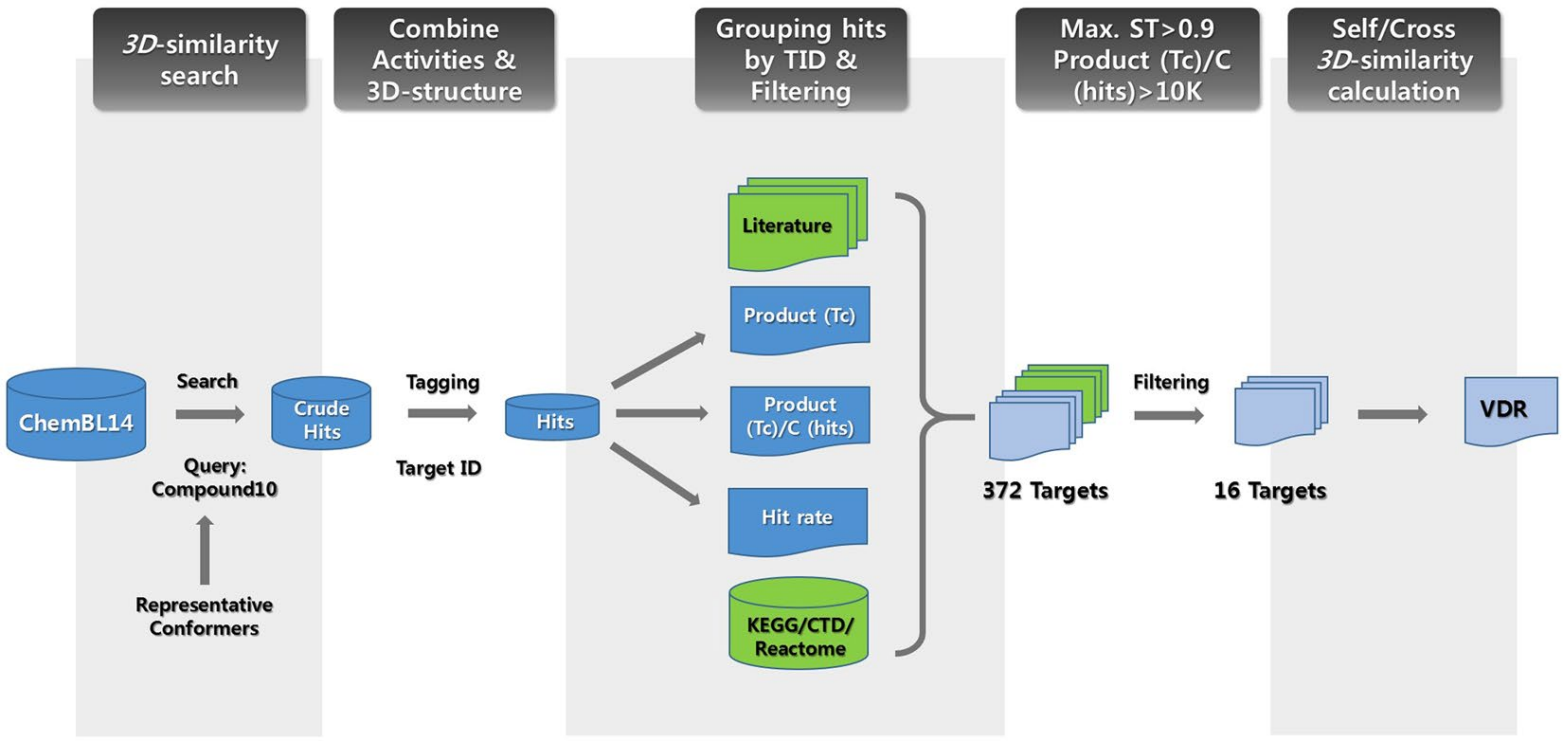
Workflow from the analysis of 3D-similarity screening data to target selection.

According to the workflow shown in Fig. 10, the screened 3D-similar compounds with multiple conformers (crude hits) were analysed for target profiling. One conformer with the highest Tc score (Tanimoto combo score) in each hit was tagged with ChEMBL target number and grouped. Among the total 1159 targets, the subcellular fractions, cell lines, tissues, and organisms were excluded to produce 973 targets. In the ligand group of each target, the next four values were calculated: (1) the multiplication of Tc score of every hit within one target; Product (Tc), (2) the number of ligands with similarity above the Tc threshold (Tc = 1.0); C (hits), (3) inverse of Product (Tc), (4) Product (Tc) divided by C (hits), (5) hit rate, and so on.

Based on the experimental results (inhibitory effect on LPS-induced NO production and cell viability), it was assumed that the target profiling of compound **10** has a high correlation with three keywords (LPS, NOS, and NO). From KEGG, Reactome, and CTD, 372 target proteins in the network of LPS, NO, and NOS were collected and combined with the information of their ligands (ChEMBL tagged) among the hit compounds of 3D-screening. The related targets (372 proteins for 216 ChEMBL compounds as their ligands) could be filtered under the condition of “Product (Tc)/C (hits)” value (threshold >10,000), producing 55 targets for 25 ChEMBL compounds as their ligands (Supplementary Table 2). With the maximum Shape Tanimoto score of their ligands (threshold of Max ST >0.9), the filtering of the 55 targets afforded 22 targets as the candidate target proteins of scaffold **1**, and the total number of ligands (with the maximum Tanimoto score of >0.9) of the 22 targets was only five. Among the 22 targets, even though CYPs also are very important proteins in drug interaction, they are not generally considered as attractive targets. So they were excluded from the target profile in our study and the ligand of CYP was also excluded from the five hit compounds (Table 4).

**Table 4:** *3D* chemo-centric predicted target profiling of the compound **10** from ChEMBL database: TOP16 targets^a^.

Target^b^	TC	ST	CT	RefT	RefC	FitT	FiTC	CS	M.TC	SD	P/C(hit)	C (hit)	C (total)
**VDR**	**1**.**68**	**0**.**97**	**0**.**90**	**1**.**02**	**0**.**95**	**1**.**09**	**0**.**97**	**1**.**83**	**1**.**15**	**0**.**08**	**9**.**6E + 14**	**298**	**19729**
*ALL1*	1.68	0.97	0.85	1.00	0.96	1.06	0.98	1.82	1.14	0.07	9.9E + 26	537	40513
*BAT8*	1.68	0.97	0.76	1.02	0.96	1.06	0.98	1.83	1.14	0.07	1.1E + 67	1243	84623
*AML1*	1.68	0.97	0.75	0.99	0.95	1.09	0.95	1.83	1.14	0.07	2.1E + 06	151	6705
*TDP1*	1.68	0.97	0.75	1.01	0.95	1.09	0.96	1.70	1.14	0.07	1.2E + 08	181	10887
*LMN1*	1.68	0.97	0.75	0.99	0.96	1.07	0.98	1.83	1.13	0.07	2.4E + 24	504	33982
*ALOX15B*	1.68	0.97	0.75	0.99	0.96	1.05	0.98	1.78	1.15	0.08	3.3E + 04	110	6559
*ERAB*	1.68	0.97	0.75	1.00	0.93	1.05	0.98	1.78	1.14	0.07	2.0E + 08	186	14801
*MAPK1*	1.68	0.97	0.75	0.99	0.94	1.09	0.98	1.78	1.14	0.07	2.6E + 11	240	15420
*HTT*	1.68	0.97	0.73	0.99	0.94	1.06	0.97	1.83	1.13	0.07	1.0E + 12	271	17391
**ESR2**	**1**.**28**	**0**.**90**	**0**.**92**	**0**.**99**	**0**.**96**	**1**.**06**	**0**.**96**	**1**.**78**	**1**.**15**	**0**.**08**	**3**.**8E + 04**	**111**	**3727**
**ESR**	**1**.**28**	**0**.**90**	**0**.**92**	**1**.**00**	**0**.**96**	**1**.**09**	**0**.**96**	**1**.**78**	**1**.**15**	**0**.**09**	**2**.**0E + 05**	**123**	**4820**
*CBX*	1.25	0.91	0.90	1.02	0.97	1.07	0.98	1.83	1.14	0.06	4.1E + 79	1475	87650
*JHDM3A*	1.25	0.91	0.85	1.02	0.96	1.07	0.98	1.83	1.14	0.06	6.5E + 44	827	47579
*ALDC*	1.25	0.91	0.79	1.02	0.96	1.07	0.98	1.83	1.14	0.06	6.3E + 57	1093	70450
*OPRD*	1.23	0.94	0.63	1.01	0.94	1.05	0.94	1.78	1.15	0.07	1.0E + 10	203	4889

^a^Description of scores: TC = the maximum value of Tanimoto scores combined with ST and CT, ST = the maximum value of Shape Tanimoto, CT = the maximum value of Colour Tanimoto, RefT = the maximum value of reference shape Tversky, RefC = the maximum value of reference colour Tversky, FitT = the maximum value of fitmol shape Tversky, FiTC = the maximum value of fitmol colour Tversky, CS = the maximum value of the combined scores, M.TC = the mean of Tanimoto scores combined with ST and CT, SD = the standard deviation of Tanimoto scores combined with ST and CT, P/C = product value P divided by the number of ligands C(hit), a product (P: multiplying) of Tanimoto scores combined with ST and CT, C(hit) = the number of ligands (C: count) with similarity above a TC threshold (TC = 1.0), C(total) = the number of ligands on each target in ChEMBL database; ^b^Target abbreviations: *VDR*, vitamin D receptor; *ALL1*, menin/histone-lysine *N*-methyltransferase; MLL, *BAT8* histone-lysine *N*-methyltransferase; H3, lysine-9 specific 3; *AML1*, runt-related transcription factor 1/core-binding factor subunit beta; *TDP1*, tyrosyl-DNA phosphodiesterase 1; *LMN1*, prelamin-A/C; *ALOX15B*, arachidonate 15-lipoxygenase type II; *ERAB*, endoplasmic reticulum-associated amyloid beta-peptide-binding protein; *MAPK1*, MAP kinase ERK2; *HTT*, huntingtin; *ESR2*, oestrogen receptor beta; *ESR*, oestrogen receptor alpha; *CBX*, chromobox protein homolog 1; *JHDM3A*, lysine-specific demethylase 4A; *ALDC*, aldehyde dehydrogenase 1A1; *OPRD*, delta opioid receptor.

**Figure 11:**
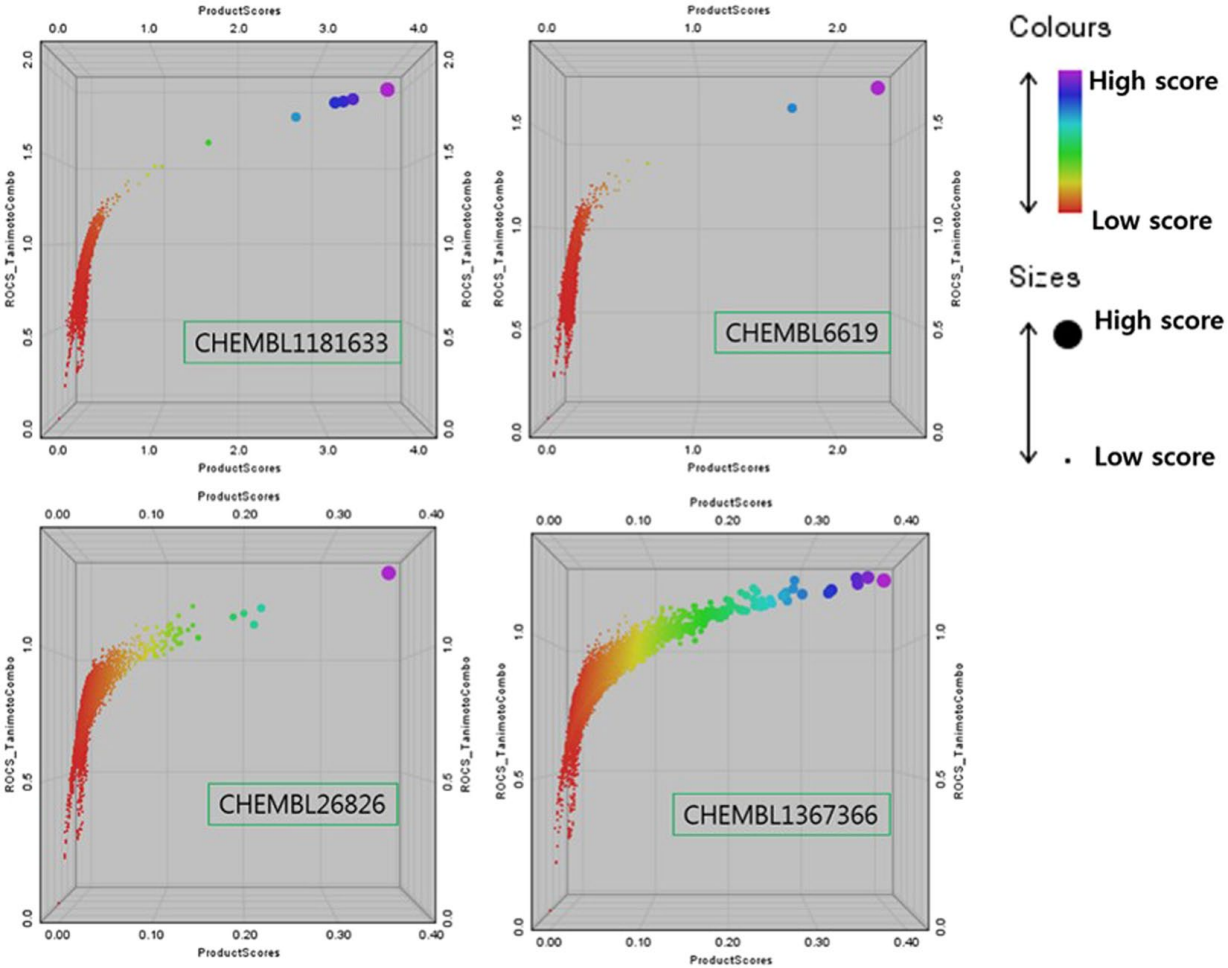
3D scatter plots for three pairwise similarity scores between the entire set of VDR in ChEMBL and four query molecules: (**a**) CHEMBL1181633 (ligand of VDR), (**b**) CHEMBL6619 (ligand of ESRs), (**c**) CHEMBL26826 (ligand of OPRD), and (**d**) CHEMBL1367366 (ligand of CBX, ALDC, and JHDM3A) (x-axis: PS resulting from the multiplication of seven scores, y-axis: combined Tanimoto scores of shape with colour, z-axis: combined Tversky scores of shape with colour).

After the exclusion of six CYPs with their ligands, the four hit compounds as a query were selected for the self/cross-similarity calculation between the query and each CHEMBL ligand of the targets indicated by the query. In the relative frequency histograms of pairwise similarity score between the query and DB ligands (Supplementary Figure 1), the similarity distribution of VDR ligands to CHEMBL1181633 (a real ligand of VDR) did not show a significant difference with the distributions of “VDR ligands” to three non-VDR ligand query molecules in the statistical parameters. However, the top 10 ranked similar VDR ligands to CHEMBL1181633 showed significantly higher Tanimoto scores than each of the top 10 compounds compared to CHEMBL6619 (ligand of ESRs), CHEMBL26826 (ligand of OPRD), and CHEMBL1367366 (ligand of CBX, ALDC, and JHDM3A) in the 3D scatter plot (Fig. 11).

In particular, the PS (product score) was generated by multiplying a total of six shape and colour scores from the three metrics (Tanimoto, Reference Tversky, and Fit Tversky) and ComboScore. Among the general operators to fuse scores, multiplication is sensitive to the change in each value less or more than score 1 for PS to maximize the difference between the VDR query (CHEMBL1181633) and others^35,36^. The reason why only the top similar compounds are able to discriminate the real VDR ligand with non-ligands is proposed next: (1) the number of binding sites for VDR inhibitors may be more than the three sites reported until today^37^; (2) the properties of binding sites may be very distinct (e.g., the available length, width, and height of a binding site for ligands; electrostatic environment of pockets; and shape of binding cavity); (3) possibility of indirect regulators existing in the VDR dataset.

### Regulatory effect of compound 10 on Cathepsin D, ER-β, and VDR expression

Among the 372 target proteins obtained from the chemocentric profiling, Cathepsin D, ER-β, VDR, and *i*NOS were selected to validate our prediction. In detail, VDR was selected from the top 16 targets in the 3D screening, and ER was selected as a commonly predicted target in both the 2D and 3D search. Cathepsin D and iNOS were selected as a middle or low-scored target. Impressively, although iNOS is a target in the representative pathway of microglia-activated neuroinflammation, the ligands of NOS family (bNOS, iNOS, and nNOS) showed a very low PS, low hit rate, and low colour scores (Table 5).

**Table 5:** *3D* chemocentric prediction result of compound 10 against NOS, TNF-α, and Cathepsin D^a^.

Target^b^	TC	ST	CT	RefT	RefC	FitT	FiTC	CS	M.TC	SD	P/C (hit)	C (hit)	C (total)
*TNF-α*	1.28	0.84	0.48	1.00	0.72	0.92	0.59	0.73	1.13	0.08	3.5E-01	6	539
*nNOS*	1.11	0.73	0.40	0.86	0.74	0.92	0.48	0.78	1.07	0.03	4.1E-01	3	1021
*eNOS*	1.11	0.73	0.40	0.86	0.74	0.92	0.48	0.78	1.07	0.03	4.1E-01	3	795
*iNOS*	1.11	0.73	0.40	0.86	0.74	0.92	0.48	0.78	1.07	0.03	4.1E-01	3	977
*CPSD*	1.21	0.76	0.49	0.97	0.73	0.97	0.63	0.74	1.11	0.04	6.0E-01	26	1173

^a^Description of scores: they are identical symbols with Table 4. ^b^Target abbreviations: TNF-αN tumour necrosis factor *alpha*; *nNOS*, NO synthase, brain; *eNOS*, NO synthase, endothelial; *iNOS*, NO synthase, inducible; *CPSD*, cathepsin D.

**Figure 12:**
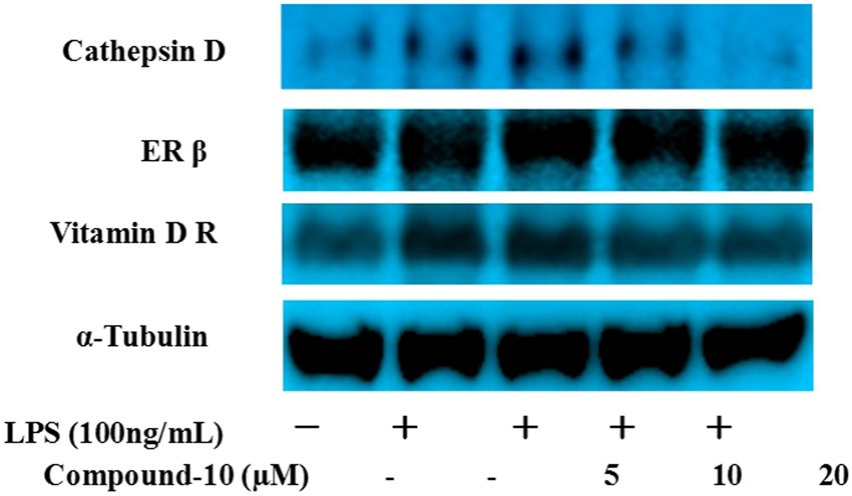
Effect of compound **10** on Cathepsin D, ER-β, and Vitamin D R protein expression in LPS-induced BV-2 cells.

**Figure 13:**
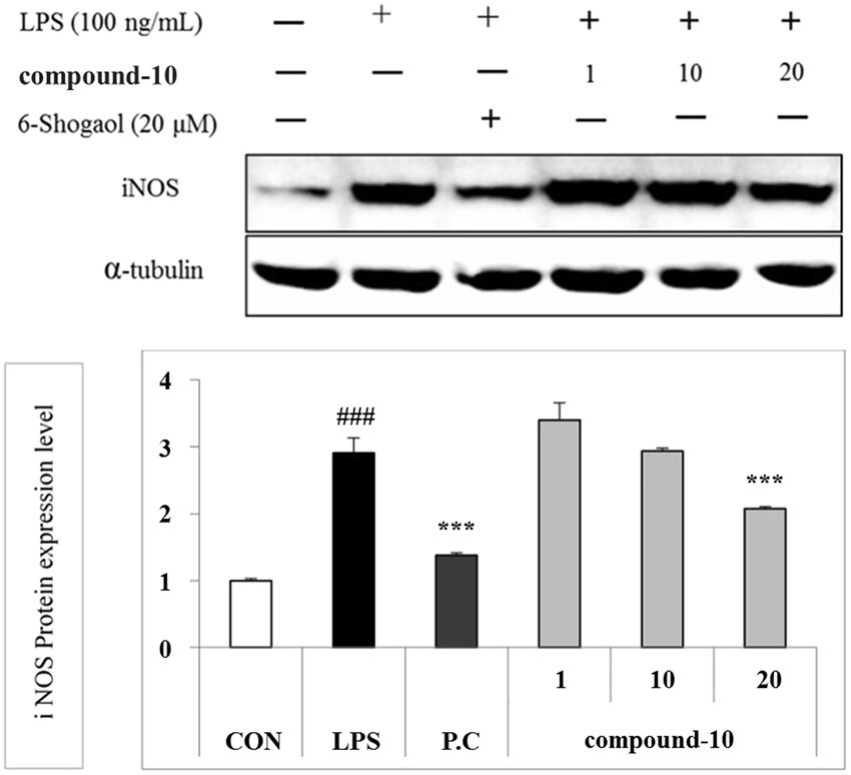
Modulation of LPS-induced iNOS expression by compound 10 in BV-2 microglial cells. BV2 cells were pretreated with the compound for 30 min and then stimulated with 100 ng/mL of LPS for 6 h. The cell lysates were extracted, and the protein levels of iNOS were analysed by Western blotting. (**A**) iNOS expression in LPS-activated BV2 cells. (**B**) Densitometric analysis of iNOS expression. α-Tubulin was used as the loading control. The figures show the representative results of three independent experiments. ^###^ *P* < 0.001 compared to the untreated control, *P < 0.05, and ***P < 0.001 compared to the LPS-treated group.

In the case of microglia-activated neuroinflammation, compound **10** concentration-dependently regulated the expression level of Cathepsin D and VDR as shown in Fig. 12. In the case of ER-β, the regulation by compound **10** did not show a distinct difference in three concentrations. In addition, the *i*NOS expression level was also regulated by compound **10** as shown in Fig. 13.

### Drug property of the unprecedented scaffold 1

According to the concept of our CHOS in the Fig. 1, various drug property of scaffold **1** was studied with promiscuity. Recently early stage filtering of undruggable compounds: (1) reactive, (2) promiscuous (frequent hitters), (3) undesired DMPK is very important before choosing lead compounds for ‘fast Go or No Go’. For the purpose, in silico filtering method like PAINS (Pan Assay Interference Compound) filter, E. Lilly medchem rules & several ‘rule of thumb’ (like Pfizer 3/75 rule, rule of five, rule of three, rule of four) have been developed^38–44^. With considering the scaffold **1** as a CNS drug, drug property was predicted from multi conformers of tested compounds (**4a**–**f**, **5**, and **10**) under Qikprop. The prediction showed solubility, polar surface area, albumin binding, BBB penetration, HERG inhibition, cell permeability (Caco-2 & MDCK), and CNS activity (Table 6, Supplementary Table 3). The potent compound **10** satisfied every standard range except for marginal log HERG value. The compound **4d** was slightly oulier from standard range among tested 8 compounds. In addition, CYP metabolism of scaffold 1 was predicted in Supplementary Tables 4 and 5. In promiscuity filtering, every compound except for the compound **10** passed 480 PAINS substructures and the compound 10 passed 479 substructures. However, dialkyl aniline, the one filtered substructure of the compound **10** showed the unsuitability for only AlphaScreen technology (as a potent quenchers of singlet oxygen), not general ‘Pan Assay Interference Compound (PAINS)’^38^.

**Table 6:** ADMET and physicochemical parameters prediction effects of compounds (4a–f, 5, and 10) using QikProp**.

No.	Nitrogen	PSA	log S^a^	log Khsa^b^	log BB^c^	CNS activity^d^	log HERG^e^	Apparent Caco-2 permeability (nm/s)^f^	Apparent MDCK permeability (nm/s)^g^	PAINS^h^
4a	0	25.09 ± 0.80	−5.23 ± 0.32	0.42 ± 0.03	−0.47 ± 0.48	−0.40 ± 0.93	−4.97 ± 0.16	9896.57 ± 33.51	5893.20 ± 21.55	pass
4b	0	25.30 ± 1.02	−7.62 ± 0.42	1.09 ± 0.02	−0.70 ± 0.46	−0.63 ± 0.78	−6.43 ± 0.14	9903.28 ± 9.63	5897.52 ± 6.20	pass
4c	0	24.28 ± 1.01	−6.31 ± 0.46	0.59 ± 0.02	−0.58 ± 0.75	−0.92 ± 1.80	−4.96 ± 0.12	9901.57 ± 14.02	10000.0 ± 0.0	pass
4d	0	42.55 ± 1.14	−10.26 ± 0.28	1.25 ± 0.08	−3.23 ± 0.01	−2.0 ± 0.0	−8.20 ± 0.35	9906.04 ± 0.0	5899.29 ± 0.0	pass
4e	0	8.43 ± 1.24	−5.06 ± 0.45	0.55 ± 0.02	−0.04 ± 0.49	0.88 ± 1.02	−5.07 ± 0.16	9900.71 ± 13.41	10000.0 ± 0.0	pass
4 f	0	29.57 ± 1.11	−3.80 ± 0.0	0.02 ± 0.03	0.079 ± 0.01	1.0 ± 0.0	−4.89 ± 0.16	5463.65 ± 142.56	3100.95 ± 87.46	pass
5	0	26.09 ± 0.92	−4.75 ± 0.14	0.24 ± 0.03	−0.69 ± 0.39	−0.71 ± 0.71	−4.66 ± 0.15	9902.90 ± 11.12	5897.28 ± 7.16	pass
10	0	16.43 ± 1.45	−5.09 ± 0.0	0.77 ± 0.03	0.22 ± 0.01	1.0 ± 0.0	−5.41 ± 0.14	9028.63 ± 287.19	5336.83 ± 183.44	^j^noticed dialkyl aniline
Standard	(0/1)	(7/200)	(−6.5/0.5)	(−1.5/1.5)	(−3.0/1.2)	−2 (inactive) ±2 (active)	(concern below −5)	(<25 poor, >500 great)	(<25 poor, >500 great)	pass

**For 95% of known drugs based on Schrödinger, USA-Qikprop v3.2 (2015) software results. Except for PAINS, every values were calculated from Qikprop. ^a^log S: log [Conformation-independent predicted aqueous solubility]; ^b^log Khsa: log [predicted binding to human serum albumin/unbound ratio]; ^c^log BB: log [predicted brain/blood partition coefficient] listed in Luco^45^ and Kelder *et al*.^46^; ^d^log HERG: log [predicted IC50 value for blockage of HERG K± channels]; ^e^CNS activity Ajay *et al*.^47^; ^h^PAINS: it was treated under J. B. Baell’s Knime workflow^38^; ^j^noticed dialkyl aniline is due to highly problematic in AlphaScreen technology (as a potent quenchers of singlet oxygen), not general ‘Pan Assay Interference Compound’^37^.

## Discussion

### Biological proof of relevance between neuroinflammation and scaffold 1

In this study, we used the chemocentric approaches to elicit plausible target profiling of in-house synthetic scaffold **1**. Although 2D approaches proposed neither highly similar ligands nor statistically significant targets, the approaches guided us to *in vitro* inflammation model. LPS-stimulated cell model is one among chemically induced typical nueroinflammation models (ex. 6-OHDA, LPS, MPTP). Microglia activation is a well-known key cellular mediator of neuroinflammation process so that murine microglia cell was chosen to treat LPS. In the MTT assay of murine BV2 cells, the cultured neuronal cells from LPS-induced neurotoxicity were rescued by scaffold **1**. In addition, the NO production in the cell was also efficiently suppressed by the compounds. When considering the SAR of scaffold 1, it seems that (1) an electron-donating substituent in an *N*-aryl group such as OMe (compound **4a**,**d**) or NMe_2_ (compound **10**) was very important for the *in vitro* neuroprotective activity; (2) an *N*-heteroaromatic ring containing a hydrogen-bonding acceptor (compound **4f**) was superior than a simple benzene ring; (3) any substituent in a tetrahydropyran ring (either electron donating or withdrawing) did not play an important role in increasing the inhibitory activity on NO production. In addition, (4) the activity disappeared by replacing the tetrahydropyran ring with a tetrahydrofuran ring (compound **5**). The presence of an *N*,*N*-dimethyl functional group on two aromatic rings (compound **10**) and pyridine moiety (compound **4f**) significantly increased the inhibitory activity of NO production. Compound **10** showed the most potent neuroprotective effect on suppressing neuroinflammation among the tested compounds; therefore, its utility was further investigated through the next steps: (1) shape-based 3D-similarity search and target profiling using the chemocentric approach and (2) experimental proof of the profile.

### Is the chemocentric target profiling of compound 10 useful?

After determining the utility of scaffold **1** in neuroinflammation, the 3D chemo-centric profiling (*in silico* prediction) was performed for further investigation, and the results were compared with *in vitro* data. Maximum similarity could satisfy the assumption of chemo-centric approach enough in the 3D screening for the compound **10** (Max.: TC = 1.78, ST = 0.97, CT = 0.95, RefT = 1.03, RefC = 0.97, FitT = 1.10, FitC = 0.982, CS = 1.85). Despite the satisfaction, our 3D chemo-centric methodology have the two limitations: (1) discrimination of direct targeting with indirect targeting, and (2) discrimination of difference within targets (withiness) with difference between targets (betweenness). First limitation can be partially originated from full usage of ChEMBL DB (including both binding assay & function assay, regardless of index). When considering benefit & loss resulting from refined ChEMBL (by activity index), we judged that scaffold diversity is more important in the 3D screening for unprecedented scaffold. Essentially, because chemo-centric assumption can guarantee a similar target but not same target, ‘refined direct binding index (ex. uM level Ki)’ was not treated in the study.

Secondly, it was challengeable to discriminate difference within targets (withiness) with difference between targets (betweenness) due to deviations of a similarity score: (1) from conformation, the deviation of a similarity score within a compound, (2) from ligand diversity, the deviation of a similarity score within a target, (3) from metric, the deviation of a similarity score within a conformer of a compound. To overcome them, the deviation from conformation was treated by (1) optimal sampling of conformational space in both query & DB chemicals^34^, and (2) choosing one conformer with maximum score after similarity calculation. In second & third deviation, rather than relying on the most similar compound, the fusion of similarity scores was tried to enhance the discrimination. In particular, multiplication of scores increased the sensitivity of values less than 1.0; thus, an outstanding value resulting from the multiplication can help us to consider more promising targets^35,36^. Therefore, the deviation from ligand diversity within a target was treated by ‘Product (Tc)/C(hits)’ in initial targets and the deviation from similarity metric was treated by ‘PS value’ in top 22 targets (Supplementary Table 2). The similarity score distribution of ligands within the c16 targets was compared by PS values. The PS value provided (1) the number of extreme similar compounds, (2) distribution of shape scores, and (3) distribution of colour score at a glance as shown in Fig. 11.

*In silico* and *in vitro* data were compared in Cathepsin D, ER-β, VDR, and *i*NOS, selected from 372 proteins. As one curious result, high ranked VDR was linked with the experiment data. VDR is a ligand-inducible transcription factor and after (1) binding with a ligand & (2) making the complex with co-activator like SRC-1, activated VDR interacts with another nuclear receptor and plays a role in inflammation^48^. Or activated Raf–MAPK–ERK may engage in cross-talk with the classical VDR pathway to modulate gene expression including pro-inflammatory factors^49^. Regulation of protein level, itself can’t be a proof for direct binding but show us that the compound **10** reduced VDR level through undisclosed mechanisms and low level VDR can contribute to suppress inflammation in activated microglia^50^. Based on high similar VDR ligands with the compound **10**, possibility of direct binding also can’t be discarded. If the compound **10** bind to VDR, three binding regions was reported^37^. Among orthosteric binding x-ray ligands, the PDB including the most similar ligand with the compound **10** was chosen and docking of the compound **10** in the PDB (3AZ1) shows high docking score and slightly advance ligand efficient rather than the original ligand (Supplementary Figure 2)^51^. Else, after checking co-activator binding site of VDR, compound **10** can also be used as a tool compound to find a new binding site^37^.

In addition, the eventual proteins (PTGS1/2) for the biosynthesis of PGE-2 and proteins modulated by PGE-2 action such as ER, MAPK, and PKC in several KEGG pathways showed reasonable “Product(TC)/C(hit)” and PS. Thus, our prediction also matched well with the experimental data, the suppressed PGE-2 production. Despite the low similarity between NOS inhibitors and compound **10**, compound **10** exhibited concentration dependent regulation of iNOS level in microglia-activated neuroinflammation. Thus, our chemocentric approach predicted the targets ER-β, and VDR of compound **10** (true positive with high scores: TC, P(Tc)/C(hits), PS). The regulation of Capthesin D, TNF-α, and iNOS was also predicted by compound **10** (true positive with low scores). The low-scored target prediction can be explained as follows: (1) insufficient ligand information in the current DB (in the case of TNF-α), and (2) low relative frequency of a similar scaffold in diverse scaffolds of ligands in the current DB (in the case of iNOS).

## Conclusion

As a part of our CHOS project, the target profiling of an unprecedented drug scaffold **1**, was investigated. First, anti-inflammatory effect of the scaffold **1** in neuronal cell was studied after the motivation from 2D-similarity based chemocentric approach. Second, 3D-screening for the compound **10** showing the most potent neuroprotective effects produced similar chemicals (as multi conformers) through the Gaussian-based similarity calculation of the implicit sampled multi-conformers. KEGG/Reactome/CTD limited the target profiles into 372 targets. Fusion of TC scores and PS (multiplication of multi-scores) of the hits improved the discrimination on the targets. Third, protein level of IL-6, TNF-α, ER-β, VDR, Capthesin D, and iNOS and PGE-2 production were concentration-dependently regulated by the compound **10**. Among them, the chemocentric target prediction successfully predicted the ER-β and VDR targeting of compound **10** and could not predict the regulation of TNF-α and iNOS by compound **10** with a strong confidence. In addition, our prediction also explains the suppression of PGE-2 level. In the near future, mechanistic investigation of the promising targets of scaffold **1** such as VDR will be performed to optimize the utility of the scaffold.

## Methods and Materials

### Chemistry

#### The general synthesis of the compound 4 (4a–f) and the compound 5

To a stirred solution of a starting material **2 or 3** (0.1 mmol) in CHCl_3_ (0.6 mL), sat. NaHCO_3_ solution (0.2 mL) was added at 0 °C. After stirring for 5 min at 0 °C, TEMPO (2.8 mg, 0.018 mmol) and iodine (23 mg, 0.09 mmol) were added to the reaction mixture in the dark. Stirring was continued for 1 h. The reaction was monitored by TLC. After completion of reaction, the reaction mixture was quenched with 1:1 ratio of *sat*-NaHSO_3_ (0.1 mL) and *sat*-NaHCO_3_ (0.1 mL) at 0 °C and the reaction mixture was diluted with CHCl_3_ (10 mL), then the water layer was extracted with CHCl_3_ (3 × 5 mL) and the combined organic layer was washed with brine, and dried over anhydrous MgSO_4_. The solvent was removed under reduced pressure below 20 °C to give the crude product. The crude product was subjected to column chromatography (25% EtOAc-hexane with 1% Et_3_N as eluent) gave cyclized product **4** (30–99%) as colorless oil. Every spectra data of the product **4** is available in the supplementary information.

#### The synthesis of the compound 10

To stirred solution of compound **8** (0.05 gr, 0.196 mmol) in 5 ml of anhydrous ether was added 3,4-dihydro-2H-pyran **9** (0.036 mL, 0.392 mmol) then reaction mixture take to 0 °C and add 1 drop of TFA, the reaction mixture was stirred 4 h at rt, monitored with TLC, after completion of SM reaction mixture was cooled to 0 °C, quenched with sat. NaHCO_3_ extracted the water layer with ether two times, the combined organic layer was washed with brine and dried over Na_2_SO_4_, filtered, and concentrated. The residue was purified by flash column chromatography (10% ethyl acetate hexane with Et_3_N (1%)) to give of compound-5 (0.043 g, 65%) as pale green liquid.

#### Spectra data of the compound 10

^1^H-NMR (600 MHz, C_6_D_6_) δ 6.97 (d, *J* = 6.97 Hz, 4 H), 6.38 (d, *J* = 6.38 Hz, 4 H), 4.70 (dd, *J* = 4.71, 2.6 Hz, 1 H), 3.70–3.64 (M, 1 H), 3.14–3.04 (m, 1 H), 2.24 (s, 12 H), 1.37–1.14 (m, 6 H); ^13^C- NMR (150 MHz, C_6_D_6_) 146.9 (2 C), 138.2 (2 C), 119.6 (4 C), 114.7 (4 C), 113.8 (2 C) 87.9, 66.6, 41.0 (2 C), 40.7 (2 C), 25.5, 24.2, 22.7 ppm; IR (FT-IR) 3726, 3708, 3620, 3612, 2916, 2846, 1738, 1506, 1458, 1240, 1170, 1076, 960, 648 cm^−1^; HRMS (ESI^+^): calcd for C_21_H_29_N_3_O^+^ [M + H]^+^: 340.2380, found: 340.2405.

### 2D-Fingerprint based similarity search

As CSNAP similarity parameters, MACCS was chosen among FP2, FP3, FP4, and MACCS and it also was chosen in cluster fingerprint. To determine threshold of list, Tanimoto coefficient (Tc) cutoff was ‘0.6’ (minimum 0.6) and Z-score cutoff was 2.5 (minimum 1, p = 0.74). Database was CHEMBL20 with confidential more than 4 (confidential range: 0~9, 9 was the highest confident) & biochemical assay type. In SEA, it was conducted under the default condition (database: ChEMBL v16 with binding 10 uM, 2D-fingerprint: ECFP4).

### 3D- similarity calculation between the scaffold 1 and ChEMBL database

Before generating conformer of database, molecules with hypervalent metal complexes were removed due to problem of charge assignment under force field. Through OE-MPI (a kind of multi-processing) in KISTI supercomputer, ChEMBL (database) were treated under the condition of (1) the MMFF94 force field excluding Coulomb interactions & the attractive part of Van der Waals, (2) 15 kcal/mol as the energy window, (3) deleting hydrogen, (4) permission on generating stereoisomers, and (5) 25 acceptable number of rotatable bond. Conformers of the query were generated under the same condition except for (1) 25 kcal/mol as the energy window, (2) fixed stereoisomer. Our dynamic tree-cut algorithm resampled query conformers. Using the resampled multi-conformers (the query), similarities between the query and conformers of the ChEMBL were calculated under the OE-MPI/ROCS condition. All jobs were with the criteria: the Tanimoto combo score (cutoff = 1.0), RefTversky combo score (cutoff = 0.7), and FitTversky combo score (cutoff = 0.7).

### Chemo-centric profiling and Target picking

Using Knime, the *in silico* hit conformers (3D structure file) acquired from the 3D-similarity screening were read, sorted by dual criteria (1^st^ criterion: unique ChEMBL number of chemical, 2^nd^ criterion: Tanimoto combo score), and only one conformer (one row) with the highest Tanimoto combo score in one ChEMBL number was filtered. And then they were tagged with activity information including biological target name with unique ChEMBL number, activity values using the Knime or in-house python code calling MySQL. In addition, every value in this study were acquired through the manipulation in Knime.

### Biological method

#### Reagents

Dulbecco’s modified eagle medium (DMEM) was purchased from Lonza (Basel, Switzerland), fetal bovine serum (FBS) and Penicillin-streptomycin (PS) were purchased from Invitrogen (Carlsbad, CA, USA). Lipopolysaccharide(LPS), 6-shogaol and N-monomethyl-L-arginine (NMMA) were purchased from Wako Pure Chemical (Osaka, Japan). ELISA kit for interleukin-6 (IL-6), tumor necrosis factor alpha (TNF-α) and prostaglandin E 2 (PGE 2) were purchased from (R&D Systems, Minneapolis MN, USA). Other all chemicals were purchased from Sigma-Aldrich (St. Louis, MO, USA).

#### Cell culture

Murine microglial cell line, BV2 cells were used to investigate the cytoprotective and antineuroinflmmatory activities of compounds. Phenotypic and functional properties of BV-2 cells are similar to reactive microglial cells. Dr. V. Bocchini at the University of Perugia (Italy) originally developed these cells. Dr. E. Choi from Korea University (Seoul, Korea) provided the BV-2 microglial cells, and these cells were maintained in DMEM supplemented with 10% FBS, 1% penicillin (1 × 10^5^ U/L), and streptomycin (100 mg/L) and kept in a humidified incubator supplied with 5% CO_2_ at a temperature of 37 °C.

#### Cell viability assay

To determine the cell viability, 3-(4,5-dimethylthiazole-2-yl)-2,5-diphenyl-tetrazolium bromide (MTT) assay was performed. Cells were cultured in 96-well plates and treated with different concentration (5 and 20 μΜ) of compounds with or without LPS. After 24 h of incubation, medium was removed and MTT solution (0.5 mg/mL) was added. The cells were incubated for an additional 1 hr. After incubation, medium was removed, and dimethyl sulfoxide (DMSO, 200 μL) was added to each well. The optical density (OD) was measured at 570 nm. The cell viability was determined by examining the ability of viable cells to decrease the yellow colored MTT to purple color formazan. The results were expressed as a percentage of the control group.

#### Measurement of nitric oxide (NO) production

The nitrite concentration in the mediun of the BV-2 cell was measured according to the Griess reaction as an indicator of NO production. To determine the NO production, BV-2 cells (4 × 10^4^ cells/well) were seeded in 96 well plates and treated with LPS (100 ng/mL) in the presence or absence of each concentration of compound (5 or 20 μΜ). After incubation at 37 °C for 24 h, 100 µL of Griess reagent containing equal volumes of 2% (w/v) sulfanilamide in 5% (w/v) phosphoric acid and 0.2% (w/v) of N-(1-naphthyl) ethylenediamine solution was added to determine nitrite production. A standard curve was created by the use of known concentrations of sodium nitrite, and absorbance was measured at 570 nm. Cell viability of the remaining cells was determined using the CCK (Cell Counting Kit, Dojindo, Kumamoto, Japan)-based colorimetric assay. For positive control, L-NMMA was used. To calculate NO concentration, sodium nitrite was used.

#### Western blot analysis

For western blot analysis, cells (1.5 × 10^6^ cells/well) were seeded in 96-well pate and stimulated with or without LPS in the presence or absence of each concentration of compound (5 or 20 μΜ) at varying time duration. For the detection of Cathepsin D, ER-β, and iNOS and vitamin D, cells were treated for 6 hr and 24 hr, respectively. After treatment, cells were washed using ice cold phosphate buffered saline (PBS). After that cells were scraped off the dishes and collected in centrifuge tube and then centrifugation was done for 5 min at 7500 rpm. For further using, cells were stored in lysis buffer. For western blot analysis, proteins obtained from cells were used. Proteins samples were loaded and separated using 8 5 sodium dodecyl sulfate polyacrymide gel electrophoresis (SDS-PAGE) and transferred to nitrocellulose membrane. The membranes were blocked with 5% skim milk for 1 h, and then washed with TBST for three times in every 10 min. After washing membrane, it was incubated overnight with primary antibodies against α-tubulin, Cathepsin D, ER-β, Vitamin D R and iNOS at 4 °C. Next day, primary antibodies were removed and membranes were washed three times with TBST like before. Membrane was incubated with respective secondary antibody for 1 h. Enhanced chemiluminescene (ECL), western blot detection reagent (GE Healthcare, Chalfont St, Giles UK) was use to visualize the the protein band. Image Lab TM software (version 5.2.1, Bio-rad) was used to measure the density of the bands.

#### Determination of TNF-α, IL-6, and PGE2 production

To determine the TNF-α, IL-6, and PGE2 productions, cells were seeded in 96 well plates (1.5 × 10^6^ cells/well) and incubated for 24 hr. After incubation, cells were treated with LPS in the presence or absence of each concentration of compound (5, 10, 20 μΜ). After an additional 24 hr incubation, supernatant was harvested and used to determine the levels of TNF-α and IL-6, and PGE2 using an ELISA development kit and competitive enzyme immune assay kit (Cayman Chemical, Ann Arbor, MI, U.S. A), respectively.

#### Statistical analysis

All data were expressed as mean ± standard error mean. One way analysis of variance (ANOVA) followed by Newman-Keuls post-hoc test using the Graph pad prism 5 software was used to determine the significance among data and significance value was set at *p* < 0.05. All experiment was performed in triplicate.

## Electronic supplementary material

Supplementary Figure & Spectra data

Supplementary Tables
