# Supplementary material for: Pharmacological use of a novel scaffold, anomeric N,N-diarylamino tetrahydropyran: molecular similarity search, chemocentric target profiling, and experimental evidence
Source: Sci Rep. 2017 Oct 2;7:12535. doi: 10.1038/s41598-017-12082-3 (PMC5624941; doi:10.1038/s41598-017-12082-3)
Supplement: Supplementary file 1 — Supplementary Figure & Spectra data [file 41598_2017_12082_MOESM1_ESM.doc]

**Pharmacological use of a novel scaffold, anomeric *N,N-*diarylamino tetrahydropyran: molecular similarity search, chemocentric target profiling, and experimental evidence**

Arramshetti Venkanna,^1^ Oh Wook Kwon, ^2^ Sualiha Afzal, ^1^ Cheongyun Jang, ^1^ KyoHee Cho, ^1^ Dharmendra K. Yadav,^1^ Kang Kim, ^1^ Hyeung-geun Park,^3^ Kwang-Hoon Chun, ^1^ Sun Yeou Kim,^1*^ Mi-hyun Kim^1*^

*^1^Gachon Institute of Pharmaceutical Science & Department of Pharmacy, College of Pharmacy, Gachon University, 191 Hambakmoeiro, Yeonsu-gu, Incheon, Republic of Korea; ^2^Natural F&P Corp. 152 Saemal-ro, Songpa-gu, Seoul, Korea; ^3^Research Institute of Pharmaceutical Science and College of Pharmacy, Seoul National University, Seoul, Republic of Korea*

^*^Author for correspondence

E-mail: [sunnykim@gachon.ac.kr](mailto:sunnykim@gachon.ac.kr) ; [kmh0515@gachon.ac.kr](mailto:kmh0515@gachon.ac.kr)

*General synthesis*

Commercial grade reagents and solvents were used without further purification. Analytical thin layer chromatography (TLC) was performed on precoated silica gel 60 F_254_ plates and visualization on TLC was achieved by UV light (254 nm) and Ninhydrine solution, and heat as developing agents. Flash column chromatography was undertaken on silica gel (230-400 mesh). ^1^H NMR was recorded on Bruker 600 MHz and chemical shifts were quoted in parts per million (ppm) referenced to the appropriate solvent peaks (7.26 ppm for CDCl3, 7.16 ppm for C_6_D_6_). The following abbreviations were used to describe peak splitting patterns when appropriate: s = singlet, d = doublet, t = triplet, q = quartet, m = multiplet, dd = doublet of doublet. J, were reported in hertz unit (Hz). 13C NMR was recorded on 125 MHz, and was fully decoupled by broad band proton decoupling. Chemical shifts were reported in ppm referenced to the center line of a triplet at 77.23 ppm of CDCl3 or at 128.0 ppm for C_6_D_6_. Optical rotations were measured on a JASCO DIP-1000 digital polarimeter. High performance liquid chromatography (HPLC) was performed on Hitachi L-7100 and L-2130 instruments using 4.6 mm × 250 mm Daicel Chiral cell OD and Chiralpak AD-H columns. Mass spectral data were obtained under the condition of Agilent LC/Q-TOP by using ESI positive method.

*Synthetic route of scheme 1 & identification of products*

**General Procedure for Cyclisation**

To a stirred solution of a starting material **2 or 3** (0.1 mmol) in CHCl_3_ (0.6 mL), sat. NaHCO_3_ solution (0.2 mL) was added at 0°C. After stirring for 5 min at 0°C, TEMPO (2.8 mg, 0.018 mmol) and iodine (23 mg, 0.09 mmol) were added to the reaction mixture in the dark. Stirring was continued for 1 h. The reaction was monitored by TLC. After completion of reaction, the reaction mixture was quenched with 1:1 ratio of *sat*-NaHSO_3_ (0.1 mL) and *sat*-NaHCO_3_ (0.1 mL) at 0°C and the reaction mixture was diluted with CHCl_3_ (10 mL), then the water layer was extracted with CHCl_3_ (3 X 5 mL) and the combined organic layer was washed with brine, and dried over anhydrous MgSO_4_. The solvent was removed under reduced pressure below 20°C to give the crude product. The crude product was subjected to column chromatography (25% EtOAc-hexane with 1% Et_3_N as eluent) gave cyclized product **4** (30-99 %) as colorless oil

**N, N-Bis-4-methoxyphenyltetrahydro-2H-pyran-2-amine: (4a)**

^1^H-NMR (600 MHz, C_6_D_6_) δ 7.15 -7.18 (m, 4 H), 6.83 -6.85 (m, 4 H), 4.88-4.90 (m, 1 H), 3.91 -3.93 (m, 1 H), 3.35 (s, 6 H), 1.52 -1.54 (m, 2 H), 1.34 -1.41 (m, 1 H), 1.20 -1.30 (m, 2 H), 1.03-2.07 (m, 1H); ^13^C- NMR (150MHz, C_6_D_6_) δ 155.8, 140.8, 125.3, 114.2, 87.6, 66.6, 54.6, 31.4, 25.4, 24.0 ppm; IR (FT-IR) 2935, 2833, 1504, 1463, 1232, 1178, 1072, 1022, 821, 748 cm^-1^ ; Yield: 70%; white oil; HRMS (ESI^+^):calcd for C_19_H_23_NO_3_^+^ [M+H]^+^: 314.1750, found: 314.1103.

**N,N-Bis-4-methoxyphenyl-4-phenyltetrahydro-2H-pyran-2-amine: (4b)**

^1^H-NMR (600 MHz, C_6_D_6_) δ7.14 -7.16 (m, 2H), 7.07 -7.09 (m, 2H), 7.00 -7.03 (m, 2H), 6.93 -6.95 (m, 2H), 6.78 -6.83 (m, 5H), 4.96 -4.98 (m, 1H), 3.97 -4.00 (m, 1H), 3.37 -3.41 (m, 1H), 3.31 (s, 6H), 2.54 -2.59 (m, 1H), 1.87 -1.90 (m, 1H), 1.59 -1.65 (m, 1H), 1.44 -1.51 (m, 1H), 1.30 -1.34 (m, 1H); ^13^C- NMR (150MHz, C_6_D_6_) δ 32.87, 39.15, 54.64, 65.99, 87.73, 114.27,125.45, 126.25, 127.84, 127.97, 128.46, 140.79, 145.25, 155.96 ppm; IR (FT-IR) 2997, 2951, 2933, 2834, 1504, 1239, 1179, 1063, 1034, 963, 821, 735, 699 cm^-1^ ; Yield 89%; White oil; HRMS (ESI): calcd for C_25_H_27_NO_3_ [M+H]: 389.1875, found: 389.1991.

**3, 3, 4, 4, 5, 5-Hexafluoro-N, N-bis-4-methoxyphenyltetrahydro-2H-pyran-2-amine: (4c)**

^1^H-NMR (600 MHz, C_6_D_6_) δ 7.10-7.13 (m, 4 H), 6.69-6.72 (m, 4 H), 5.10-5.14 (m, 1 H), 3.49-3.55 (m, 1 H), 3.25 (s, 6 H), 2.89-2.97 (m, 1 H); ^13^C- NMR (150MHz, C_6_D_6_) δ 157.7, 140.3 114.9, 88.0, 78.0, 70.7, 65.2, 63.6, 55.2, 34.4, 32.7, 29.7, 25.5, 23.5, 14.7 ppm; IR (FT-IR) 3725, 3704, 3623, 3599, 2917, 2850, 2359, 1738, 1508, 1466, 1242, 1171, 1075, 970, 672, 649 cm^-1^ ; Yield 45%; white oil; HRMS (ESI^+^): calcd for C_19_H_23_NO_3_^+^ [M+H] ^+^: 422.1191, found: 422.1195.

**3,4,5-Trisbenzyloxy-*N,N*-bis-4-methoxyphenyltetrahydro-2H-pyran-2-amine:(4d)**

^1^H-NMR (600 MHz, CDCl_3_) δ 7.13 -7.35 (m, 15 H), 6.92 -6.95 (m, 4 H), 6.76 -6.78 (m, 4 H), 4.68 -4.70 (m, 1 H), 4.61 -4.65 (m, 1 H), 4.49 -4.52 (m, 1 H), 4.43 -4.45 (m, 1 H), 4.33 -4.34 (m, 1 H), 3.94 -4.04 (m, 2 H), 3.84 -3.90 (m, 1 H), 3.78 (s, 6 H), 3.73 -3.83 (m, 3 H), 3.65 -3.70 (m, 1 H); 13C- NMR (150MHz, C_6_D_6_) δ 154.3, 142.5, 138.2, 128.3, 127.82, 122.37, 114.6, 79.40, 78.8, 76.14, 74.19, 73.40, 71.66, 60.85, 55.60, 54.38 ppm; Yield 80%; red colour liquid; IR (FT-IR) 3062, 3030, 2918, 2850, 2383, 2359, 2348, 2342, 2326, 1737, 1508, 1243, 1036, 752, 698 cm^-1^ ; HRMS (ESI^+^): calcd for C_19_H_23_NO_3_^+^ [M+H] ^+^: 631.2832, found: 631.2832. Chiral Pak column: AD-H, eluent = Hex: 2-PrOH 70:30, Flow rate = 1mL/min; [α] _D_ ^20^ = -0.17 (c 0.90, CHCl_3_); [α]_D_ ^20^ = +0.17 (c 0.90, CHCl_3_).

**4-Fluorophenyl-N-phenyltetrahydro-2H-pyran-2-amine: (4e)**

^1^H-NMR (600 MHz, C_6_D_6_) δ 7.12 -7.19 (m, 2 H), 6.97-6.99 (m, 4 H), 6.87-6.90 (m, 1 H), 6.76-6.80 (m, 2 H), 4.77 -4.79 (m, 1 H), 3.80 -3.83 (m, 1 H), 3.19-3.24 (m,1 H), 0.9 -1.45 (m, 6 H); 13C- NMR (150MHz, C_6_D_6_) δ 160, 147.8, 141.48, 129, 121.47, 120.92, 115.4, 86.70, 66.69, 29.86, 25.21, 23.85 ppm; Yield 99%; White oil; IR (FT-IR) 2918, 2850, 2381, 2359, 2348, 2308, 1736, 1595, 1506, 1496, 1396, 1216, 1073, 1024, 755, 691 cm^-1^ ; HRMS (ESI^+^): calcd for C_17_H_18_FNO^+^ [M+H] ^+^: 272.1451, found: 272.1442.

**N-(4-Methoxyphenyl)-N-(tetrahydro-2H-pyran-2-yl)pyridin-3-amine: (4f)**

^1^H-NMR (600 MHz, C_6_D_6_) δ 8.58 (s, 1 H), 8.26 (s, 1 H), 7.10-7.07 (m, 2 H), 6.95-6.93 (m, 1 H), 6.95-6.93 (m, 1 H), 6.79-6.77 (m, 1 H), 6.73-6.70 (m, 2 H), 4.73 (dd, *J* = 4.7, 2.7 Hz, 1 H), 3.80-3.77 (m, 1 H), 3.28 (s, 3 H), 3.20-3.16 (m, 1 H), 1.43-1.42 (m, 1 H), 1.33-1.32 (m, 2 H), 1.16-1.13 (m, 3 H); ^13^C- NMR (150MHz, C_6_D_6_) 158.5, 145.2, 140.6, 139.3, 135.4, 131.5 (2 C), 122.8, 122.6, 114.5 (2 C), 86.1, 66.5, 54.5, 30.9, 25.1, 23.6 ppm; IR (FT-IR) 2942, 2836, 1510, 1473, 1223, 1177, 1071, 1018, 812, 748 cm^-1^ ; Yield: 78%; white oil; HRMS (ESI^+^):calcd for C_17_H_20_N_2_O_2_^+^ [M+H]^+^: 285.1524, found: 285.1219.

**N, N-Bis 4-methoxyphenyl tetrahydrofuran-2-amine: (5)**

^1^H-NMR (600 MHz, C_6_D_6_) δ 7.29 -7.31 (m, 4 H), 6.74 -6.81 (m, 4 H), 4.69 -4.71 (m, 1 H), 3.82 -3.86 (m, 1 H), 3.76 3.79 (m, 1 H), 3.29 (s, 6 H), 1.87 -1.91 (m, 1 H), 1.29 -1.44 (m, 3 H); ^13^C-NMR (150 MHz, C_6_D_6_) δ 156, 141.3, 125.8, 114.2, 95.4, 87.4, 66, 59.8, 54.6, 40.2, 39, 31.9, 20.1, 17.2 ppm; Yield 31%; Dark brown oil; HRMS (ESI): calcd for C_18_H_21_NO_3_ [M+H]: 299.1521, found: 299.1521.

*Synthetic route of scheme 2 & identification of products*

**N^1^-(4-(Dimethylamino) phenyl)-N^4^, N^4^-dimethylbenzene-1, 4-diamine (8):**

The oven dried resalable schlenk tube was charged with of Pd_2_(dba)_3_ (0.092 g, 0.101 mmol), X-Phos (0.096 g, 0.202 mmol), NaO^t^Bu (0.339 g, 3.35 mmol) then add degassed anhydrous Toluene (5 mL), degas through pumping Argon, then add N^1^, N^1^-dimethylbenzene-1,4-diamine 4-bromo-N, N-dimethylaniline (0.500 gr, 2.52 mmol) and N^1^, N^1^-dimethylbenzene-1, 4-diamine (0.377gr, 2.77 mmol) the schlenk tube was capped with a septum and then evacuated and back filled with Argon, the resulting mixture stirred for 10 min at rt. The septum was replaced with a Teflon screwcap. The schlenk tube was sealed and the mixture was stirred at 100-120 for 16 h, monitored with TLC , after completion of aryl halide the reaction mixture was cooled to room temperature, diluted with DCM (15 mL) , filtered through celite. Filtrate was concentrated and purified by column chromatography with 25% ethyl acetate- hexane gave compound (0.592 g, 92%) as pale brown crystal.

^1^H-NMR (600 MHz, Actone-*d6*) δ 6.93 (d, *J* = 6.93 Hz, 4 H), 6.72 (d, *J* = 6.72 Hz, 4 H), 6.41 (brs 1 H), 2.84 (s, 12 H); ^13^C- NMR (150MHz, Actone-*d6*) 145.5 (2 C), 136.4 (2 C), 118.7 (4 C), 114.5 (4 C), 40.87 4 C) ppm, m.p. 115-116 °C (Hexanes).

**N^1^-(4-(dimethylamino)phenyl)-N^4^,N^4^-dimethyl-N^1^-(tetrahydro-2H-pyran-2-yl)benzene-1,4-diamine (10):**

To stirred solution of compound -3 (0.05 gr, 0.196 mmol) in 5 ml of anhydrous ether was added 3,4-dihydro-2H-pyran (**4**) (0.036 mL, 0.392 mmol) then reaction mixture take to 0^o^C and add 1 drop of TFA, the reaction mixture was stirred 4 h at rt, monitored with TLC , after completion of SM reaction mixture was cooled to 0^o^C, quenched with sat. NaHCO_3_ extracted the water layer with ether two times, the combined organic layer was washed with brine and dried over Na_2_SO_4_, filtered, and concentrated. The residue was purified by flash column chromatography (10% ethyl acetate hexane with Et_3_N (1%)) to give of compound-5 (0.043 g, 65%) as pale green liquid.

^1^H-NMR (600 MHz, C_6_D_6_) δ 6.97 (d, *J* = 6.97 Hz, 4 H), 6.38 (d, *J* = 6.38 Hz, 4 H), 4.70 (dd, *J* = 4.71, 2.6 Hz, 1 H), 3.70-3.64 (M, 1 H), 3.14-3.04 (m, 1 H), 2.24 (s, 12 H), 1.37-1.14 (m, 6 H);^13^C- NMR (150MHz, C_6_D_6_) 146.9 (2 C), 138.2 (2 C), 119.6 (4 C), 114.7 (4 C), 113.8 (2 C) 87.9, 66.6, 41.0 (2 C), 40.7 (2 C), 25.5, 24.2, 22.7 ppm; IR (FT-IR) 3726, 3708, 3620, 3612, 2916, 2846, 1738, 1506, 1458, 1240, 1170, 1076, 960, 648 cm^-1^ ; HRMS (ESI^+^): calcd for C_21_H_29_N_3_O^+^ [M+H]^+^: 340.2380, found: 340.2405.

Spectra pictures of unpublished compounds

**^1^H NMR spectrum of 4f (600 MHz, C_6_D_6_)**


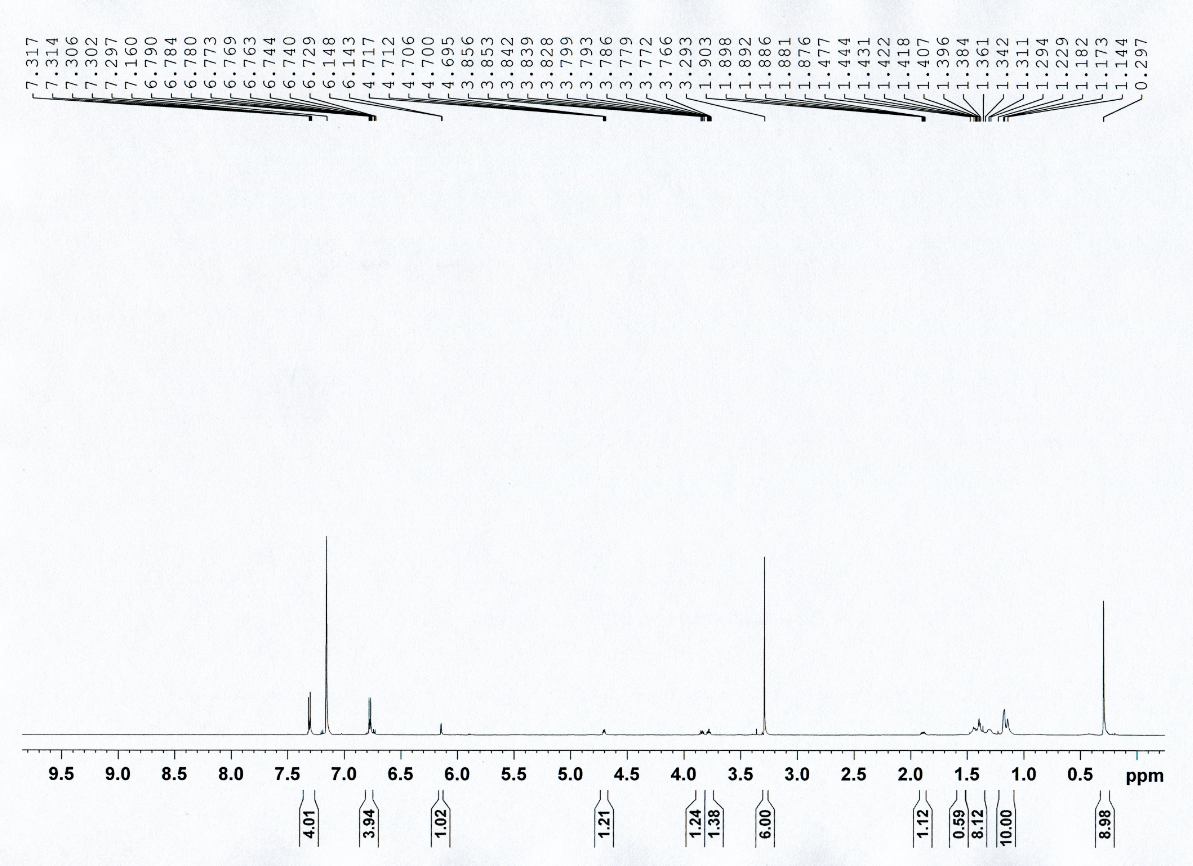
**^13^C NMR spectrum of 4f (150 MHz, C_6_D_6_)**

**^1^H NMR spectrum of 5 (600 MHz, C_6_D_6_
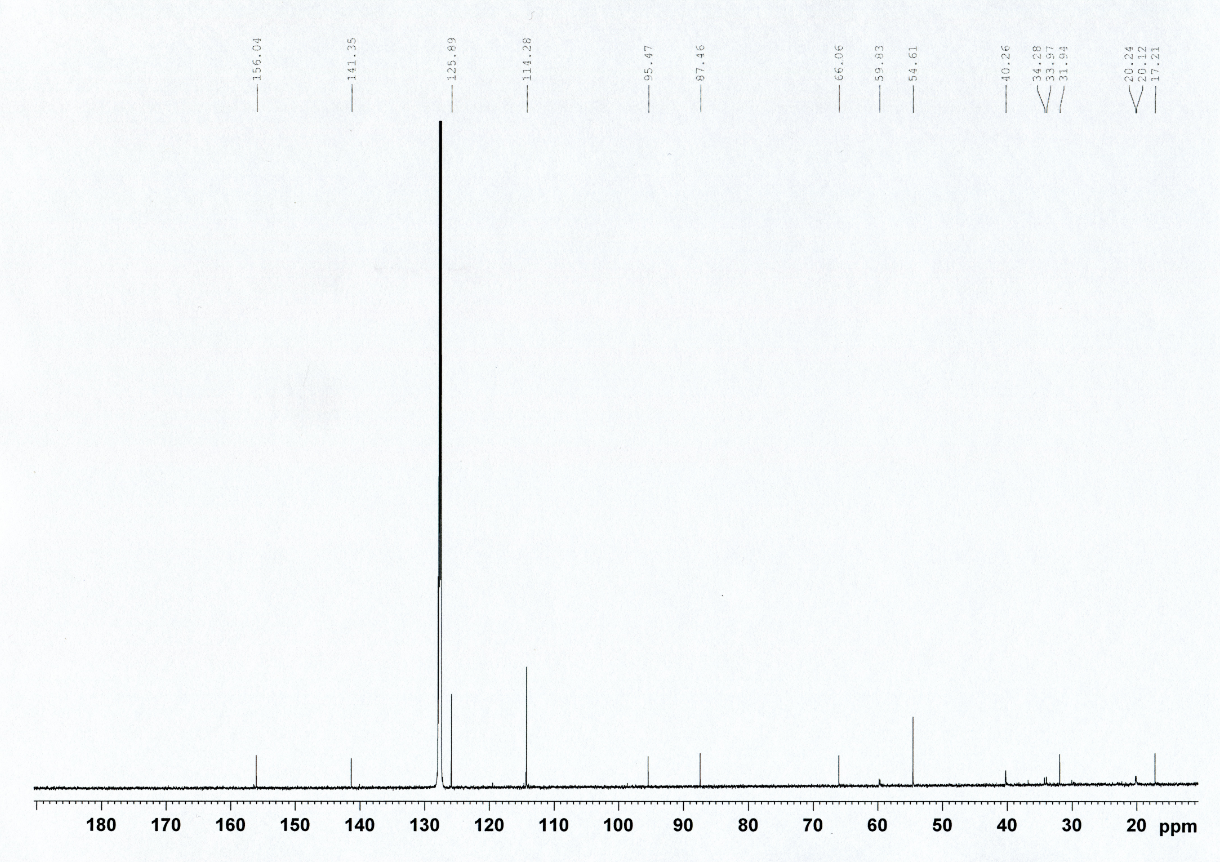
)**

**^13^C NMR spectrum of 5 (150 MHz, C_6_D_6_)**

**^1^H NMR spectrum of 8 (600 MHz, Acetone-d6)**

**^13^C NMR spectrum of 8 (150 MHz, Acetone-d6)**

**^1^H NMR spectrum of 10 (600 MHz, C_6_D_6_)**

**^13^C NMR spectrum of 10 (150 MHz, C_6_D_6_)**

Supplementary Fig 1. The relative frequency histograms of pairwise similarity score between the query (four query) and DB ligands (VDR ligands in ChEMBL): the query is (a) CHEMBL1181633 (ligand of VDR), (b) CHEMBL6619 (ligand of ESRs), (c) CHEMBL26826 (ligand of OPRD), and (d) CHEMBL1367366 (ligand of CBX, ALDC, and JHDM3A)

(a)


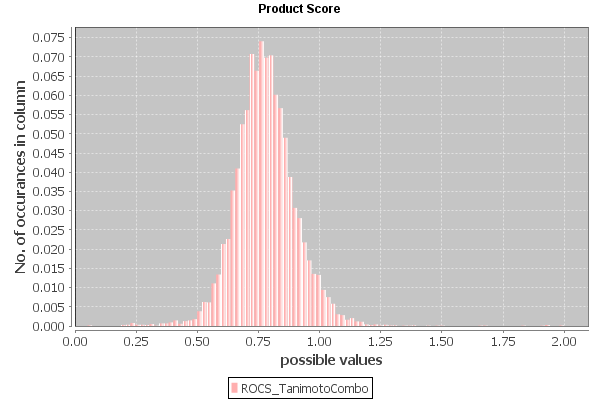


(b)


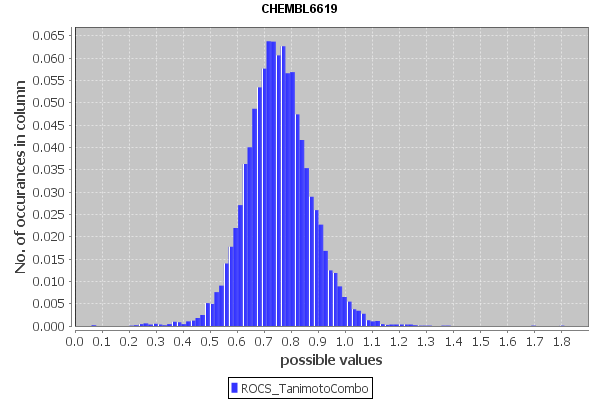


(c)


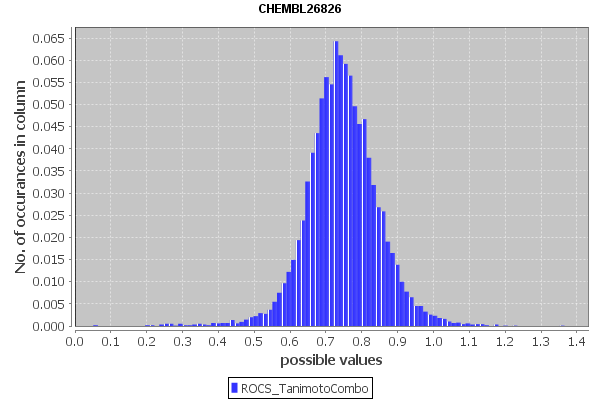


(d)


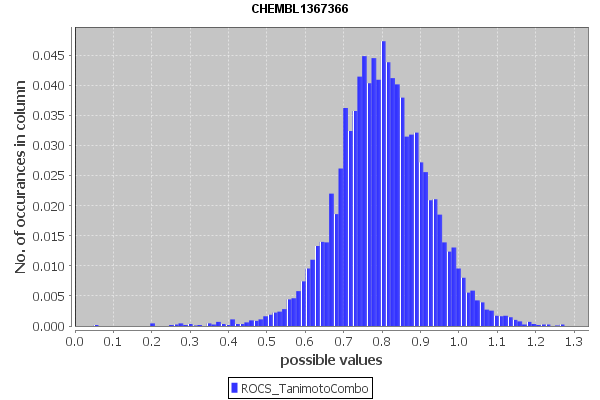


Supplementary Fig 2.


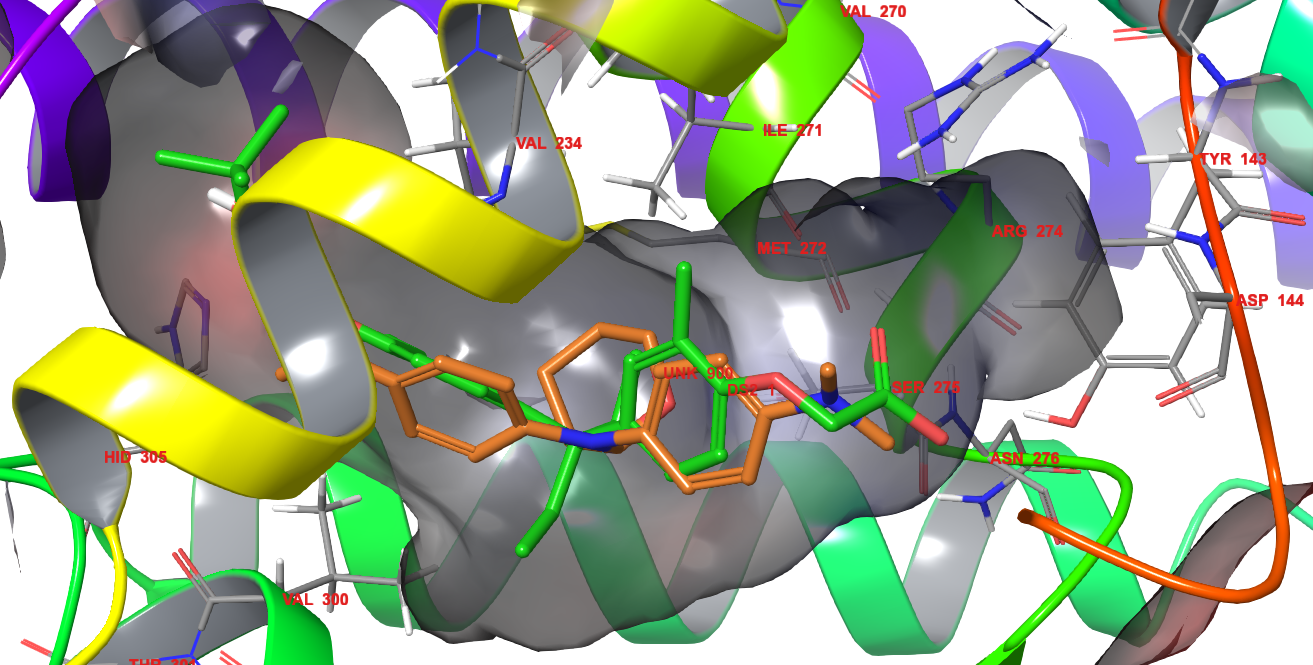


Best docking pose of compound **10** on vitamin D receptor (PDB: 3AZ1, green : original ligand (ID: DS2, vitamin D(3) analogue), orange : BNDS-A compound). The Docking score and Ligand efficiency of BNDS-A compound are -8.691 and -0.348 respectively, which is similar to the value of original ligand (-9.597, -3.000). To get this result, 101 initial BNDS-A conformers were generated, and the parameters using for docking were set as follows. (Include input conformation = check, Scoring function = SP, Enhance conformational sampling by 4 times, maximum number of minimization steps = 200, RMS deviation is less than 0.2A, write out at mose 100 poses per ligand, number of poses per ligand to include 500) For the original ligand used in the comparison, only scoring function was performed without docking. The other setting parameters were the same.
